# Subtle variation in sepsis-III definitions markedly influences predictive performance within and across methods

**DOI:** 10.1038/s41598-024-51989-6

**Published:** 2024-01-22

**Authors:** Samuel N. Cohen, James Foster, Peter Foster, Hang Lou, Terry Lyons, Sam Morley, James Morrill, Hao Ni, Edward Palmer, Bo Wang, Yue Wu, Lingyi Yang, Weixin Yang

**Affiliations:** 1 Mathematical Institute, University of Oxford, Oxford, UK; 2 Department of Mathematics, University College London, Room 603, 25 Gordon St, London, WC1H 0AY UK; 3 Bloomsbury Institute of Intensive Care Medicine, University College London, London, UK; 4 The Alan Turing Institute, London, UK; 5 Center for Precision Psychiatry, Massachusetts General Hospital, Boston, MA USA; 6 Department of Mathematics and Statistics, University of Strathclyde, Glasgow, UK; 7 Department of Mathematical Sciences, University of Bath, Bath, UK

**Keywords:** Diseases, Health care, Signs and symptoms

## Abstract

Early detection of sepsis is key to ensure timely clinical intervention. Since very few end-to-end pipelines are publicly available, fair comparisons between methodologies are difficult if not impossible. Progress is further limited by discrepancies in the reconstruction of sepsis onset time. This retrospective cohort study highlights the variation in performance of predictive models under three subtly different interpretations of sepsis onset from the sepsis-III definition and compares this against inter-model differences. The models are chosen to cover tree-based, deep learning, and survival analysis methods. Using the MIMIC-III database, between 867 and 2178 intensive care unit admissions with sepsis were identified, depending on the onset definition. We show that model performance can be more sensitive to differences in the definition of sepsis onset than to the model itself. Given a fixed sepsis definition, the best performing method had a gain of 1–5% in the area under the receiver operating characteristic (AUROC). However, the choice of onset time can cause a greater effect, with variation of 0–6% in AUROC. We illustrate that misleading conclusions can be drawn if models are compared without consideration of the sepsis definition used which emphasizes the need for a standardized definition for sepsis onset.

## Introduction

The rise in comprehensive electronic health record systems (EHRSs) has enabled the application of state-of-the-art machine learning (ML) models in predictive diagnostics^1–3^. In this context, machine learning typically focuses on developing models to maximise predictive accuracy against a given target clinical definition. The aim for the research community is to develop and critique models to find the best solutions for prediction. However, little attention is paid to small variations in these definitions used to define a predictive target when operationalised within the EHRS. Consequently, variations in clinical definitions, and their interpretation within an EHRS, lead to severe difficulty in fair comparisons of predictive methods across the literature. An interesting research question that arises from this current practice is how robust are the conclusions drawn from a comparison of different studies if there exist subtle differences in target definition? We seek to explore this, in the context of predicting sepsis onset time, by quantifying the impact that variations in onset time definitions have on a suite of methods (which are chosen from tree-based methods, deep learning, and survival analysis methods to cover popular and performant methods used for sepsis onset prediction).

Sepsis—a heterogeneous syndrome characterized by infection-induced organ dysfunction^4^—is a global health concern. In 2017 alone, sepsis was estimated to affect nearly 50 million people worldwide, resulting in 11 million deaths^5^. EHRSs do not contain a structured “ground truth” identifying sepsis. The sepsis-III definition operationalizes sepsis within an EHRS as an increase in the sequential organ failure assessment (SOFA) score^6^ by two or more, in the presence of suspected or confirmed infection. In lieu of a gold standard label, it is a requirement for studies in this domain that clinical data are labelled with a “sepsis phenotype”^7^. In the sepsis-III study^8,9^, this phenotype was developed using the coincident administration of antibiotics and taking of blood cultures as a proxy for “suspicion of infection.” This allowed the labelling of patients who are likely to have sepsis, and so created a target definition for statistical models. Building on this definition, machine learning-based early warning systems have been studied for numerous clinical tasks, such as detection of sepsis^10–13^ and septic shock^14–16^.

The sepsis-III definition does not explicitly define the sepsis onset time, leading to different interpretations when this is of primary interest^8,11,17–22^. A recent systematic review^23^ of machine learning methods for sepsis prediction highlighted the inconsistencies in sepsis definition across different papers and that most of the associated code is not publicly available. See also^24^ for a review and discussion on the controversy relating to sepsis definition from the clinical perspective. This lack of a precise onset time, together with the absence of verifiable implementations of machine learning models, prevents effective comparison between performance of different methods.

From the sepsis-III definition, we can see that it is ambiguous whether the onset should be defined as the time of organ failure, the time of the suspicion of infection, or the earlier of these two events. All three are used across the literature. We consider these three competing interpretations of sepsis definition by evaluating them with a suite of advanced models on the MIMIC-III dataset^25^. Each definition, depending on when organ dysfunction or suspected infection is identified, has a distinct clinical interpretation^11,17,18^. MIMIC-III contains high frequency data for patients admitted to ICU. We note that although this has some limitations, this is a dataset frequently utilized for sepsis prediction tasks and can highlight the issues relating to subtle variations in sepsis label construction. We found significant differences in predictive performance between definitions, confirming the need for precise and verifiable methods, to allow comparisons to be made.

This study is accompanied by code implementing a pipeline for the development of early warning scores and their evaluation, including the use of signature methods for time series analysis. All code used in this study has been made publicly available^26^, to allow further development and comparisons. This manuscript is prepared in accordance with the RECORD statement; the extension to STROBE for research based on routinely collected data^27^.

## Materials and methods

### Determining the onset of sepsis: ${\varvec{t}}_{{{\mathbf{sepsis}}}}$

Determining the onset time of sepsis ($t_{{{\text{sepsis}}}}$), requires identification of the onset of suspected infection ($t_{{{\text{suspicion}}}}$) and the time at which the SOFA score deteriorates by at least two points ($t_{{{\text{sofa}}}}$). We align to the sepsis-III definitions^9^ as closely as possible and describe any necessary deviations.

We used the drawing of blood cultures and the administration of antibiotics, within proximity to one another, as surrogates for clinical suspicion of infection. We identified the times that blood cultures were taken ($t_{{{\text{cult}}}}$) and that a course of antibiotics was first administered ($t_{{{\text{abx}}}}$). As patients typically took multiple doses of the same antibiotic, subsequent doses of the same antibiotic were classed as being in the same course, as long as consecutive doses were administered within two days of each other. Only the initial time of each course of antibiotics was used to look for clinical suspicion. We required at least two doses of any type of antibiotics to be administered within a 96-h period for a $t_{{{\text{abx}}}}$ to count towards a suspicion of infection. One-off prophylactic antibiotics were not included.

**Figure 1:**
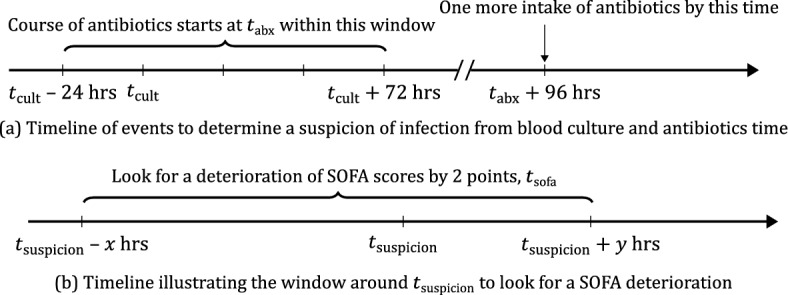
Timelines to determine the time of suspected infection (panel **a**) and the sepsis onset time (panel **b**). We name the interval around ${\text{t}}_{{{\text{suspicion}}}}$ as the “SOFA window” where {x, y} denotes the time in hours before and after ${\text{t}}_{{{\text{suspicion}}}}$ that is used for the detection of a change in SOFA score.

A valid suspicion of infection required that if the antibiotic was given first, the culture must be obtained within 24 h, or that if the culture was taken first, the antibiotic must have been administered within 72 h. Once $t_{{{\text{cult}}}}$ and $t_{{{\text{abx}}}}$ had been identified with appropriate proximity, the onset of suspected infection ($t_{{{\text{suspicion}}}}$) was defined as the first occurrence in this pair (Fig. 1a).

To identify organ dysfunction attributable to infection, we required an increase in the SOFA score of least two points (at time $t_{{{\text{sofa}}}}$) in an interval around $t_{{{\text{suspicion}}}}$ (Fig. 1). We call this interval the “SOFA window” described by the quantities {*x*, *y*} as in Fig. 1b. We calculated $t_{{{\text{sofa}}}}$ as the first time that the SOFA score was two above its initial value. This is a popular approach for the early detection problem^11,18^.

For any given SOFA window, three competing onset times for sepsis (${t}_{{\text{sepsis}}}$) exist. These are labelled as **H1**, **H2** and **H3** and defined as follows:

- **H1:** $t_{{{\text{sepsis}}}} = t_{{{\text{sofa}}}}$, sepsis onset occurs at the onset of salient organ dysfunction.
- **H2:** $t_{{{\text{sepsis}}}} = t_{{{\text{suspicion}}}}$, sepsis onset occurs at the onset of suspicion of infection.
- **H3:** $t_{{{\text{sepsis}}}} = {\text{min}}\left( {t_{{{\text{sofa}}}} ,t_{{{\text{suspicion}}}} } \right)$, sepsis onset occurs at the earlier of these two events.

### Data resource

We used the MIMIC-III dataset^25^ to assess the impact of these definitions on the prediction of sepsis onset. MIMIC-III comprises de-identified patient-level data from over 40,000 patients that stayed in intensive care units (ICUs) between 2001 and 2012 at the Beth Israel Deaconess Medical Center, Boston, Massachusetts (USA). The MIMIC-III dataset was approved by the Institutional Review Boards of Beth Israel Deaconess Medical Center and the Massachusetts Institute of Technology.

Information on antibiotics was recorded infrequently prior to 2004. Since the year of hospitalization was removed during the de-identification process, we excluded all patients recorded under the “CareVue” system, which was known to be in operation only until 2008. Only those patients recorded under the “MetaVision” system is retained. Further details can be found at^25^.

Data were split into training (85%) and test (15%) sets by stratified sampling based on sex, age, intensive care length of stay, and whether the patient ever received invasive ventilation. This split was performed prior to applying further exclusion criteria, and only one hospital visit was retained per patient. We chose this split to balance between having enough data in the training set to calibrate the models (with cross validation) and having a reasonable size of test set (to reduce variance in results).

Granular information on the time of antibiotic administration and organ dysfunction was not available prior to ICU admission (for example, prescriptions only have data for date prescribed) hence precise sepsis onset time cannot be identified. Following^28^, we excluded patients who were prescribed antibiotics before their entry to ICU. Patients attending the cardiothoracic surgical ICU were excluded since their requirements for organ support are likely to produce false positive labels in the sepsis phenotype. Elective surgical patients were excluded as they are often prescribed prolonged antibiotic prophylaxis pre-arrival to the ICU and have, in general, a low risk of developing sepsis in their index ICU admission. We excluded patients whose length of stay in the ICU was shorter than four hours or longer than twenty days, to avoid the development of sepsis prior to ICU admission, or bias from atypical visits. Patients missing all vital sign data were removed since these likely represent cases with data quality issues.

**Figure 2:**
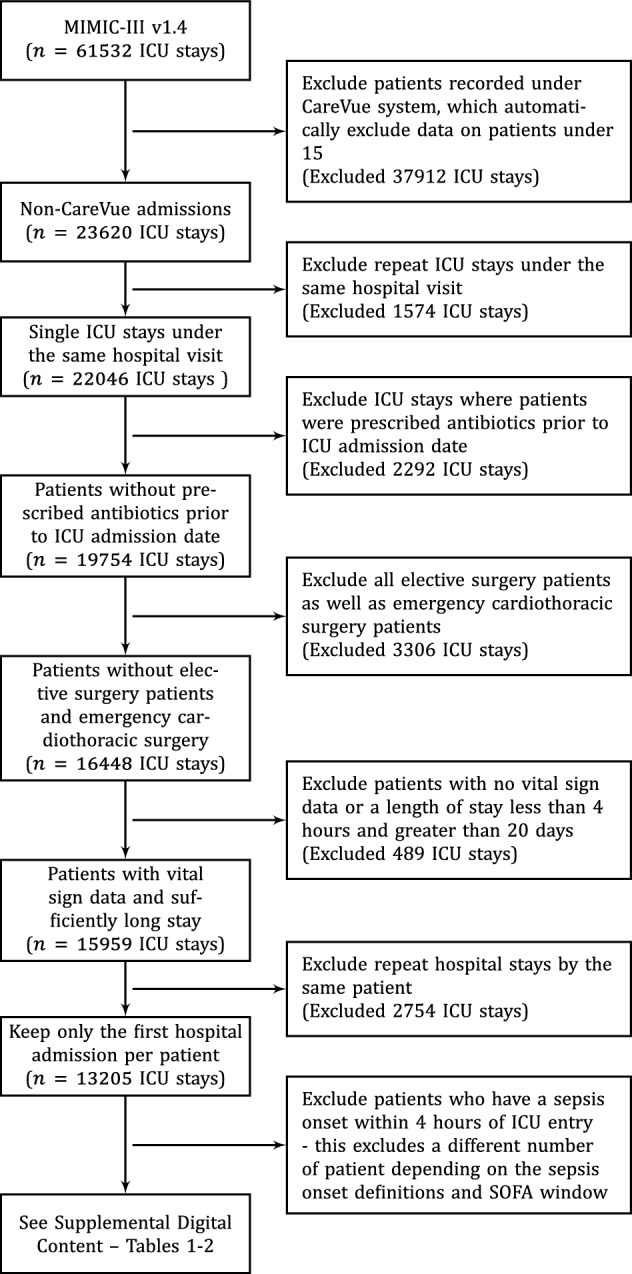
Flow Study flow diagram. Different analysis cohorts are produced depending upon the sepsis onset definitions and SOFA windows applied. These are detailed in Supplemental Digital Content—Table 1 and Supplemental Digital Content-Table 2.

Finally, we excluded patients who developed sepsis within four hours following entry to the ICU. Given the different interpretations of sepsis onset defined above (**H1**–**H3**) and SOFA windows of varying size, excluding patients who developed sepsis within four hours of entry to the ICU excluded a different number of patients depending on these choices. A study flow diagram is given in Fig. 2. See Appendix A for further information on the training and test set after exclusions.

### Modelling

Using MIMIC-III data from the described cohort, we fitted several models to assign an hourly risk score, given all salient patient information up to the present, which indicates the likelihood a patient will develop sepsis over some pre-defined prediction horizon (*T* hours). For each definition of sepsis onset time (**H1**–**H3**) we evaluated three models which have been previously used in sepsis prediction tasks: a light gradient boosting machine (LGBM)^19^, a modified Cox proportional hazards model (CoxPHM)^29^ and the long short-term memory (LSTM) neural network^16^. These are representative models from tree-based models^30–32^, survival analysis^33–36^, and deep-learning based models^37–39^ respectively and further, these three classes cover a wide range of models used for this application. In particular, an LGBM model won the 2019 PhysioNet Computing in Cardiology Challenge on early prediction of sepsis^21^. Demonstrating how the performance of this winning model compares against other popular models on different definitions emphasizes the need for transparency in model comparison. See Appendix D and E for further details on the models and how we selected the hyperparameters for our study. We investigated the impact of subtle variations in sepsis definition by looking at the changes in performance and the relative ranking of these models.

**Figure 3:**
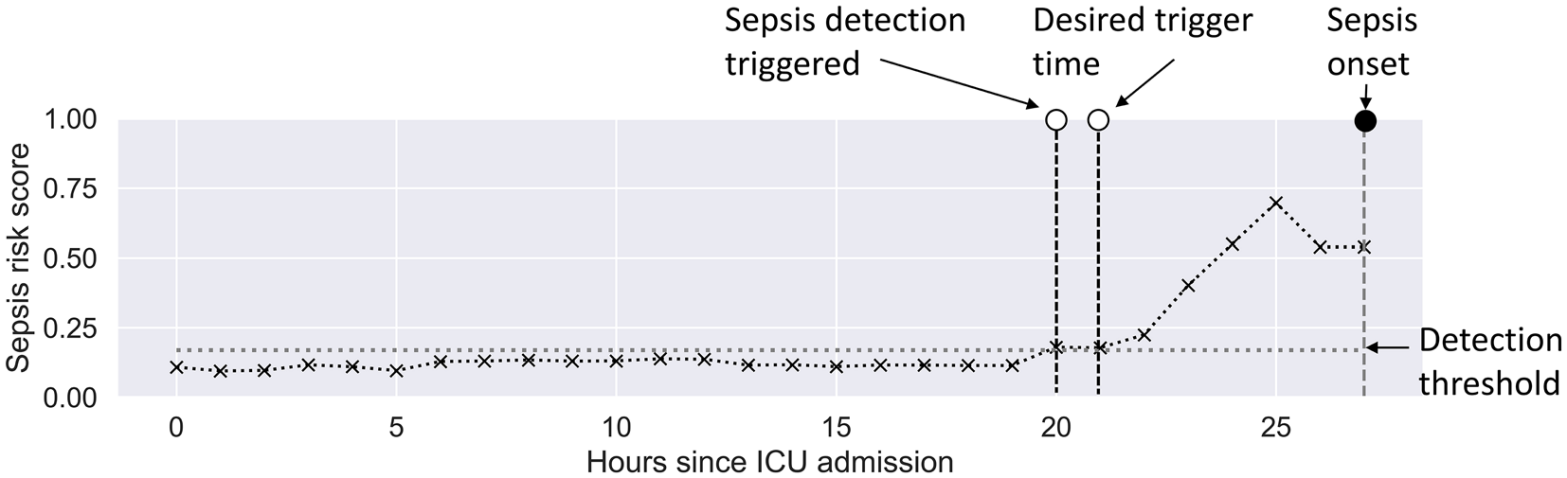
An illustrative example of converting sepsis risk scores to binary sepsis. The dotted black line with “x” markers indicates the risk score, while the horizontal dotted line indicates the chosen cutoff threshold to determine binary labels. In this case, the model predicted a positive sepsis label for the first time at 20 h after ICU admission. Here the prediction horizon T is set at 6. This indicates that the model is designed to predict 6 h ahead of the true sepsis onset time, which in this case is 21 h into the stay.

Risk scores predicted by each model were converted into binary predictions by selecting a threshold risk score above which a patient would be classified as septic (Fig. 3). This threshold was set to achieve a sensitivity of 85% on the training set, as was used in a prior study^11^. We refer to this task as the real-time prediction task (See Appendix B for the precise problem formulation).

Models were trained on observations prior to the sepsis onset time (${t}_{{\text{sepsis}}}$), to ensure that the models were predictive, rather than replicating the chosen sepsis definition. Each model was trained against each definition of sepsis onset time (**H1**–**H3**) providing nine models for each set of {*x*, *y, T*}.

Models were fitted with $38$ raw and $75$ derived predictor variables, similar to those used in Morrill et al.^13,14,16^, though we did not use end tidal carbon dioxide, as it is typically unavailable in MIMIC-III. Raw variables were in four broad categories: demographics (e.g. age, sex), vital signs (e.g. heart rate, pulse oximetry), laboratory results (e.g. bicarbonate, pH), and admission information (e.g. time since admission to hospital/ICU). A full list of raw variables used is found in Supplemental Digital Content—Table 6. In addition to these raw variables, we derived time-dependent features to use in our models (Supplemental Digital Content—Table 7). Time varying features were processed using a rolling window and signature transformation; these capture key geometric and temporal properties of timeseries data^21,40,41^. Further discussion of feature extraction is found in Appendix C.

As our goal is real-time sepsis prediction, models were trained to optimize prediction of patient labels averaged over all patients and observation times. The hyperparameters of each model were tuned with $5$-fold cross-validation performed against training data. Models with the best performing hyperparameters were subsequently refitted against the full training set to obtain final model parameters before evaluation on the test set. All methods were performed in accordance with the relevant guidelines and regulations.

### Evaluation

To evaluate model performance, we calculated the area under the receiver operator characteristic curve (AUROC, or c-statistic) of each model. This describes the predictive performance of models, averaged over all patients and observation times.

### Sensitivity analysis

Three main sensitivity analyses were considered to evaluate the sensitivity of the models and sepsis onset definitions. First, different sizes of SOFA window were considered. Second, we retrained all models for the **H1** and **H2** sepsis onset definitions, but with ICU admissions excluded using the strictest exclusion criteria from **H3**. This allowed us to examine the differences between **H1** and **H2** with the same cohort (as **H3**), and so isolate any discrepancies to the sepsis definitions and models, and not to differences in cohorts (input data). Last, microbiological samples other than blood cultures were included in the definition of $t_{{{\text{sofa}}}}$.

## Results

Following our study exclusions, between 737 and 1861 ICU admissions were available for model training, with 140 and 317 admissions in the test sets, depending upon the sepsis onset definition and SOFA window under investigation (Supplemental Digital Content—Table 1 and Table 2). Baseline characteristics for the cohort with SOFA window {24, 12} are shown in Supplemental Digital Content—Table 3.

### Prediction task

We compared the real-time prediction performance of LGBM, LSTM and CoxPHM models for each sepsis onset definition. Table 1 shows the performance of definitions in terms of test metrics (AUROC, specificity and accuracy), with **H2** yielding consistently higher scores than **H1** or **H3**, independent of the model chosen. The earliest sepsis onset time (**H3**) consistently gives the lowest performance scores. It is interesting to note that the variation in performance across sepsis criteria (0–6% AUROC) exceeds the variation in performance across models (1–5% AUROC).

**Table 1:** Summary of AUROC, specificity, accuracy of LGBM, LSTM and CoxPHM for the real-time prediction on the test set.

	AUROC	Specificity	Sensitivity	Accuracy
	**H1**			
LGBM	0.832 [0.823,0.842]	0.784	0.723	0.725
LSTM	0.805 [0.796,0.815]	0.596	0.811	0.601
CoxPHM	0.799 [0.789,0.809]	0.546	0.850	0.553
	**H2**			
LGBM	0.869 [0.862,0.876]	0.761	0.808	0.806
LSTM	0.856 [0.848,0.863]	0.718	0.813	0.721
CoxPHM	0.844 [0.836,0.852]	0.672	0.831	0.677
	**H3**			
LGBM	0.829 [0.818,0.840]	0.768	0.713	0.714
LSTM	0.793 [0.781,0.805]	0.524	0.831	0.529
CoxPHM	0.780 [0.769,0.793]	0.490	0.840	0.496

Here the SOFA window is {$\mathrm{24,12}$} and prediction horizon ${\text{T}}=6$. The mean [lower 95% confidence, upper 95% confidence] of AUROC with non-parametric bootstrapped confidence intervals drawn from 100 resamples are provided Specificity and accuracy are calculated based on that the sensitivity on the training set was chosen to be 85%.

**Figure 4:**
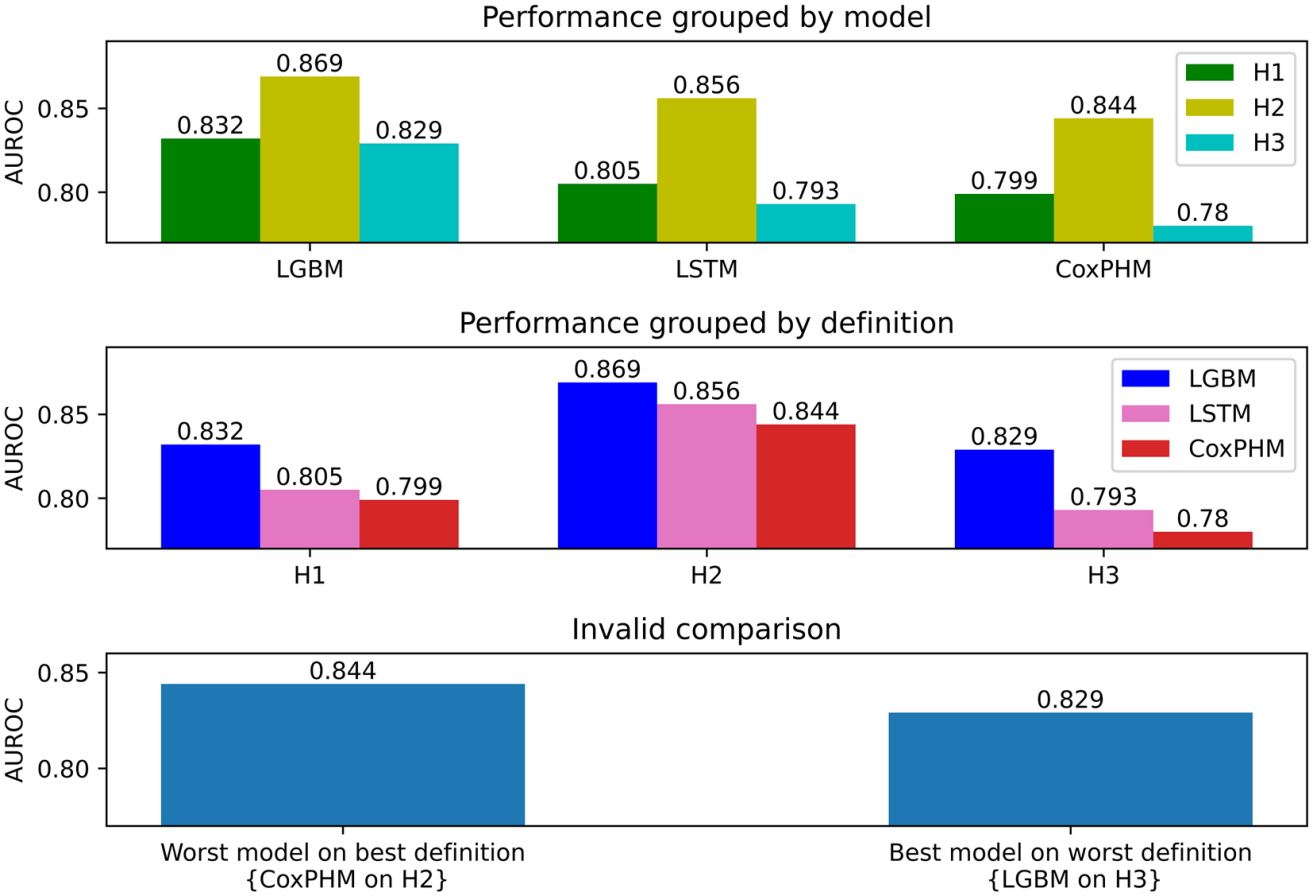
Highlights in the differences of AUROC for the SOFA window {24,12}. (Top panel) AUROC when models are evaluated on the test set, grouped by model. (Middle panel) AUROC when models are evaluated on the test set, grouped by sepsis onset definition. (Bottom) An illustration of an invalid comparison between models when the underlying sepsis definition is different. Here the best performing model on the worst definition, that is, LGBM on **H3** has a lower AUROC than the worst performing model on the best definition, namely CoxPHM on **H2**.

The results are represented visually in the two top subplots of Fig. 4. We see that LGBM outperforms the others in that it has the highest AUROC for each fixed sepsis definition. However, in the bottom subplot, it can be seen that the best model on the worst performing definition (LGBM on **H3**) performs worse than the worst model on the best definition (CoxPHM on **H2**). This emphasizes the point that without full transparency on the design choice, fair comparison across models is not possible. Naive comparison across different definitions may result in misleading conclusions.

Full details of the evaluation, including the effects of changes in SOFA window and time horizon are detailed in Appendix F of Supplemental Digital Content—Tables 9 and 10. The evaluation of the real-time prediction method is consistent with model evaluation in the literature. However, we can infer other interesting outcomes by looking at predictions made on each patient. Further discussion can be found in Appendix G.

### Sensitivity analyses

#### Impact of SOFA deterioration observation window

**Figure 5:**
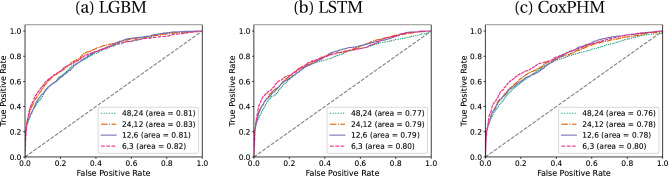
ROC plots for real-time prediction at different SOFA windows for ${\text{T}}=6$ and definition **H3** on the test set for (**a**) LGBM, (**b**) LSTM and (**c**) CoxPHM model.

All models were robust to variations of the SOFA window. Figure 5 demonstrates that, despite optimizing the models’ hyperparameters using the SOFA window {24,12}, the receiver operating characteristic (ROC) curves of the prediction from each model are very close for different SOFA windows under **H3**. For similar plots of the other two definitions see Supplemental Digital Content—Fig. 3.

#### Impact of strict exclusion criteria

Table 2 shows that the performance of each model under **H1** and **H2** decreases with the use of the strict exclusion criteria (that is, excluding patients who develop sepsis, according to **H3**, within the first four hours). While the models continue to be ranked similarly under these exclusion criteria, the performance variation of each model across definitions become smaller, especially LSTM and CoxPHM. This suggests that the superior performance observed earlier under **H1** and **H2** is largely due to the differences in patient exclusion—it appears the task of predicting sepsis is more difficult under **H3** than **H1** and **H2** principally due to the exclusion of more patients who develop sepsis soon after admission.

**Table 2:** AUROC scores of the three models (LGBM/LSTM/CoxPHM) on the test data using main extraction method and the other data exclusion choices for the real-time prediction with SOFA window {$\mathrm{24,12}$} and ${\text{T}}=6$.

	H1	H2	H3
	LGBM/LSTM/CoxPHM	LGBM/LSTM/CoxPHM	LGBM/LSTM/CoxPHM
Main extraction method	0.832/0.805/0.798	0.869/0.856/0.844	0.829/0.793/0.780
Strict exclusion	0.832/0.796/0.785	0.851/0.805/0.795	0.829/0.793/0.780
Other cultures	0.816/0.792/0.778	0.847/0.837/0.816	0.801/0.756/0.751

#### Impact of culture selection

The definition of suspected infection was broadened to include specimens other than blood culture. We note that in this case, the performance across all definitions and models shown in Table 2 decreased.

## Discussion

Using representative models from both classical statistics and machine learning, we have demonstrated that model performance in predicting sepsis onset was markedly sensitive to subtle variations in onset definitions. This performance impact was at times more pronounced than the gains from the different models themselves. Taking the LGBM model (which was consistently the best model for fixed definitions) to predict sepsis under **H3** gives a lower AUROC than using the CoxPHM (which performed worst for each fixed definition) to predict sepsis with **H2** definition. If these results were reported across different papers and compared without consideration of the underlying sepsis definitions used, we would get misleading conclusions.

There was a consistent ranking of onset definitions across all models, with the order **H2**, **H1** and **H3**. This characterization of performance is consistent with one prior large database study^17^. In the context of predictive modelling, this does not imply the models targeting the **H2** definition are any better at predicting the true sepsis condition of the patient. Rather, the models perform better at predicting the onset time defined using this definition and, in particular, using data after the corresponding exclusion criteria have been applied. This highlights the significance that a change in onset definition can have on model performance with everything else fixed. We note that these results are not evidence for whether **H2** should be chosen as the standard definition.

Significant variation in the application of sepsis onset definitions exists in the prediction literature, including examples of **H1**^11^, **H2**^9^ and **H3**^10,12,14,15^. As a definition applied to retrospective data, **H1** appears to be the most clinically meaningful, given that sepsis is defined as a “life-threatening organ dysfunction caused by a dysregulated host response to infection”^9^. Thus, conditional on there being a confirmed suspicion of infection, the point of deterioration in organ function would best reflect the moment a patient becomes septic. However, we have demonstrated the sensitivity of a wide range of high-fidelity models to these target definitions.

There is no current consensus on the best SOFA window around $t_{{{\text{suspicion}}}}$ to look for an increase in SOFA. Seymour et al.^8^ used several SOFA windows, ranging from 3 to 48 h prior to $t_{{{\text{suspicion}}}}$ and 3–24 h following $t_{{{\text{suspicion}}}}$. Others have used a SOFA window from 24 h prior to 12 h post $t_{{{\text{suspicion}}}}$ ^11,17^. Our results are reassuring, as this aspect of the definition has limited impact on predictive performance, even though the cohort size changes significantly.

Finally we note that modern data science advancements, for example, extracting signature features, can provide improved techniques for signaling the likelihood of an impending event from patient observations. However, it is impossible to compare mechanisms across the literature and develop quality tools without developing standard benchmark criteria. Our study highlights the pressing need for a gold-standard sepsis phenotype for machine learning research on early sepsis detection. The current definitions, based on SOFA, have been optimized for ease of use of application at the bedside. This imposes natural limitations for advanced statistical models. For example, the SOFA score comprises laboratory results, which are typically measured only once per day. In contrast, other constituents of SOFA comprise underlying biological data which may be continuously monitored; the juxtaposition of these may reduce statistical power^42^. Improvements to sepsis criteria, both in the precision of definition and in connection with clinical practice, will allow greater contributions to flow from machine learning research.

## Conclusion

To summarize, with the availability of electronic healthcare datasets, we can conduct numerous retrospective studies to design predictive models. However when comparing the vast amount of research, the precise construction of the target label can be overlooked. Our work demonstrates that a subtle difference in sepsis criteria leads to significant variation in model performance. Under any fixed sepsis definition, our implementation of LGBM was consistently the best model whilst CoxPHM was consistently the worst. However, if we compared the performance of LGBM under the **H3** definition against CoxPHM under the **H2** definition, then we would find that the AUROC of CoxPHM is higher. If these were models presented by different papers (using different sepsis definitions), then we may inaccurately conclude that CoxPHM is the better model for sepsis prediction. The difference in the interpretation of the clinical criteria for sepsis created an issue which has not been addressed in literature.

In general, considering different methods, while ignoring variations in the criteria used to evaluate them, may lead to comparisons that draw invalid conclusions and hinder the progress to find the best predictive model. Therefore, we make the following recommendations for retrospective studies on EHRS data:

- The data management protocol should include the full details of extracting the clinical target of interest from EHRSs, to allow full reproducibility. Publication of results should typically include a link to the code used.
- A high-quality early warning system should consistently outperform other methods and be robust to the variations in data.
- A gold-standard for the clinical outcome of interest, in a format which can be extracted from EHRSs, should be established by the joint effort of the clinical community and machine learning community.

### Limitations

Owing to the availability of data assets like MIMIC, the majority of research in sepsis prediction in the ICU has been performed on cohorts that commence at the time of ICU admission, as is the case here. However, it is important to note that most sepsis cases admitted to an ICU display evidence of organ dysfunction prior to arrival in the ICU^8^. Extending this field of research to pre-ICU observational data comes with its own inherent challenges, since patients outside ICU are monitored far less frequently and will be subject to informative sampling based on their acute physiology. This highlights the importance of developing high-quality pre-ICU data as a target for future research.

Patients who received antibiotics prior to ICU admission were necessarily excluded from our study, since the MIMIC-III database does not contain granular detail on organ dysfunction prior to arrival in the ICU. It is probable that the sepsis onset time in these cases was prior to admission to the ICU, and including these patients would have unreasonably biased model fit.

Our primary aim is to investigate the discrepancy in model performance and potential risks in misleading model comparisons caused by a subtle variation on the sepsis definition. To conduct a systematic and comprehensive comparative study, we have narrowed our scope to three representative models, employing only forward-filling as our data imputation technique. This means that we explore neither the full range of predictive methods used for sepsis prediction nor the large literature of data imputation methods available. Therefore, although we have found that LGBM performs best in this analysis, we do not claim that it is the state-of-the-art model for early detection of sepsis. Moreover, we acknowledge the limitation of the forward-filling, which can be further improved by alternative imputation method for improving the predictive performance. These are important considerations when finding the best predictive model, but does not affect our conclusion that transparency and a standard sepsis definition is vital to identify such models.

### Supplementary Information

Supplementary Information.

## Data Availability

The data we used in this paper is extracted from the MIMIC-III database. Once the required training and credentials are obtained, this dataset is accessible from PhysioNet at https://physionet.org/content/mimiciii/1.4/. The MIMIC-III project was approved by the Institutional Review Boards of Beth Israel Deaconess Medical Center (Boston, MA) and the Massachusetts Institute of Technology (Cambridge, MA). Requirement for individual patient consent was waived because the project did not impact clinical care and all protected health information was deidentified.

## Code availability

Our code is publicly available at https://zenodo.org/record/5168789. We have included detailed instructions on how to obtain the exact dataset used starting from the MIMIC-III database, as well as precise instructions on running the method so that we have an end-to-end pipeline to fully replicate the results.
