# Supplementary material for: Subtle variation in sepsis-III definitions markedly influences predictive performance within and across methods
Source: Sci Rep. 2024 Jan 22;14:1920. doi: 10.1038/s41598-024-51989-6 (PMC10803347; doi:10.1038/s41598-024-51989-6)
Supplement: Supplementary file 1 — Supplementary Information. [file 41598_2024_51989_MOESM1_ESM.docx]

Online Data Supplement

# Appendix A: Data Summary

We provide further details on the impact of the exclusions. In MIMIC-III, we have information on the time of intake so we define antibiotic doses to be in the same course if they are of the same type and taken within 2 days of each other. Based on laboratory results prior to ICU admission, some patients are diagnosed with sepsis at admission. We exclude these patients from our dataset, along with those who develop sepsis within four hours of ICU admission (specific to each definition).

The number and percentage of septic patients for each septic definition in the training set and testing set are given in Table 1 and Table 2.

Table 1: The number of patients with sepsis identified in the training set stratified by different onset definitions (**H1­­–H3**) and SOFA windows (x, y). In parenthesis are the total number of patients of the cohort after exclusions (including non-septic patients) and the percentage of this cohort with sepsis.

| $x,y$ | **H1** | **H2** | **H3** |
| --- | --- | --- | --- |
| **48,24** | 1220 (7550, 16.16%) | 1861 (8191, 22.72%) | 776 (7106, 10.92%) |
| **24,12** | 1117 (7753, 14.41%) | 1733 (8369, 20.71%) | 815 (7451, 10.94%) |
| **12,6** | 941 (8135, 11.57%) | 1514 (8708, 17.39%) | 823 (8017, 10.27%) |
| **6,3** | 783 (8903, 8.79%) | 1059 (9179, 11.54%) | 737 (8857, 8.32%) |

Table 2: The number of sepsis patients (total number of patients after exclusions, proportion of sepsis patients) for different definitions with various SOFA windows $\left( x,y \right)$ on the test set.

| $x,y$ | **H1** | **H2** | **H3** |
| --- | --- | --- | --- |
| **48,24** | 243 (1351, 17.99%) | 317 (1425, 22.25%) | 164 (1272, 12.89%) |
| **24,12** | 228 (1382, 16.50%) | 290 (1451, 20.47%) | 169 (1323, 12.77%) |
| **12,6** | 183 (1432, 12.78%) | 262 (1511, 17.34%) | 157 (1406, 11.17%) |
| **6,3** | 140 (1558, 8.99%) | 166 (1584, 10.48%) | 130 (1548, 8.40%) |

Baseline characteristics for the cohort with SOFA window {24, 12} are shown in Supplemental Digital Content – Table 3.

Table 3: Baseline mean characteristics of patients identified in the training set. A comparison is drawn between those identified with sepsis according to different definitions and the patients remaining in the cohort after exclusions have been applied but who were not found to be septic (non-septic). As a result, there will be overlap between these groups in terms of patient membership, with different values caused by the exclusion criteria discussed above. The values given in the table below are the mean, with standard deviation given in brackets where measure of spread is also of interest.

| Characteristic | | **H1** | **H2** | **H3** | **Non-septic** |
| --- | --- | --- | --- | --- | --- |
| $n$ | | 1117 | 1733 | 815 | 6636 |
| Age | | 62.71 (17.7) | 63.14 (17.4) | 62.32 (17.7) | 63.13 (18.8) |
| Sex | Male | 0.60 | 0.63 | 0.63 | 0.54 |
|  | Female | 0.40 | 0.37 | 0.37 | 0.46 |
| Ethnicity | White | 0.716 | 0.707 | 0.717 | 0.717 |
|  | Black | 0.078 | 0.070 | 0.060 | 0.115 |
|  | Hispanic | 0.043 | 0.040 | 0.040 | 0.040 |
|  | Asian | 0.026 | 0.027 | 0.026 | 0.026 |
|  | Other | 0.044 | 0.051 | 0.049 | 0.031 |
|  | Unknown | 0.093 | 0.105 | 0.108 | 0.072 |
| First care unit | Coronary Care Unit | 0.128 | 0.118 | 0.140 | 0.178 |
|  | Cardiac Surgery Recovery Unit | 0.130 | 0.204 | 0.169 | 0.035 |
|  | Medical ICU | 0.307 | 0.270 | 0.209 | 0.438 |
|  | Surgical ICU | 0.210 | 0.194 | 0.231 | 0.198 |
|  | Trauma/Surgical ICU | 0.225 | 0.215 | 0.252 | 0.151 |
| Length of stay pre-ICU (hrs) | | 16.10 (41.0) | 21.70 (47.5) | 20.04 (45.8) | 7.02 (27.2) |
| ICU Length of stay (hrs) | | 129.4 (105) | 124.2 (106) | 141.0 (110) | 49.7 (46) |
| ICU Length of stay before onset (hrs) | | 26.17 (44.2) | 22.48 (37.2) | 31.80 (49.5) | N/A |
| Mortality | | 0.126 | 0.141 | 0.129 | 0.084 |
| Max SOFA score | | 6.015 (3.24) | 6.772 (3.47) | 6.449 (3.25) | 3.378 (2.56) |
| Max SOFA > 4 | | 0.624 | 0.708 | 0.686 | 0.272 |

For the real-time prediction problem, we group the data into hourly bins by clock hours. The $0^{th}$ hour for a patient is the first hour that they are in the ICU. This data is right censored at the sepsis onset time. This results in a reduced number of observations (i.e. individual time-points at which a prediction is made). Missing data are imputed by forward-filling. The resulting total number of observations (hourly for each patient) after exclusions, along with the number of observations of septic patients, is given in Table 4 for the training data and Table 5 for the test data.

Table 4: The number of total samples and septic samples of the real-time prediction on the training set, under different definitions, values of SOFA windows $\left( x,y \right)$, and prediction horizon $T$ after data processing. For each pair of $\left( x,y \right)$, the top row indicates the total number of samples for the given definition; the other rows give the number of septic samples for corresponding definition and prediction horizon $T$.

| $x,y$ | $T$ | **H1** | **H2** | **H3** |
| --- | --- | --- | --- | --- |
| **48,24** |  | 320485 | 335540 | 313598 |
|  | **12** | 11929 | 19128 | 7442 |
|  | **8** | 9580 | 15224 | 6000 |
|  | **6** | 8041 | 12574 | 5085 |
|  | **4** | 6090 | 9275 | 3875 |
| **24,12** |  | 355521 | 366203 | 352137 |
|  | **12** | 10725 | 17592 | 8090 |
|  | **8** | 8659 | 14106 | 6375 |
|  | **6** | 7315 | 11692 | 5364 |
|  | **4** | 5575 | 8641 | 4070 |
| **12,6** |  | 408266 | 415333 | 406950 |
|  | **12** | 9160 | 14710 | 8149 |
|  | **8** | 7329 | 12121 | 6447 |
|  | **6** | 6176 | 10155 | 5427 |
|  | **4** | 4700 | 7551 | 4110 |
| **6,3** |  | 490426 | 493378 | 489908 |
|  | **12** | 7588 | 9999 | 7202 |
|  | **8** | 6110 | 8309 | 5780 |
|  | **6** | 5156 | 7048 | 4869 |
|  | **4** | 3915 | 5290 | 3685 |

Table 5: The number of total samples and septic samples of the real-time prediction on the test set, under different definitions, values of SOFA windows $\left( x,y \right)$, and prediction horizon $T$ after data processing. For each pair of $\left( x,y \right)$, the top row indicates the total number of samples for the given definition; the other rows give the number of septic samples for corresponding definition and prediction horizon $T$.

| $x,y$ | $T$ | **H1** | **H2** | **H3** |
| --- | --- | --- | --- | --- |
| **48,24** |  | 57440 | 60048 | 56236 |
|  | **12** | 2353 | 3301 | 1579 |
|  | **8** | 1877 | 2619 | 1265 |
|  | **6** | 1590 | 2147 | 1077 |
|  | **4** | 1215 | 1577 | 820 |
| **24,12** |  | 63526 | 65065 | 62862 |
|  | **12** | 2164 | 3067 | 1660 |
|  | **8** | 1746 | 2446 | 1310 |
|  | **6** | 1489 | 2007 | 1107 |
|  | **4** | 1140 | 1477 | 840 |
| **12,6** |  | 71545 | 72692 | 71279 |
|  | **12** | 1795 | 2653 | 1590 |
|  | **8** | 1414 | 2150 | 1240 |
|  | **6** | 1192 | 1768 | 1036 |
|  | **4** | 910 | 1305 | 780 |
| **6,3** |  | 86687 | 87055 | 86587 |
|  | **12** | 1375 | 1660 | 1303 |
|  | **8** | 1091 | 1335 | 1029 |
|  | **6** | 918 | 1111 | 859 |
|  | **4** | 700 | 830 | 650 |

# Appendix B: Problem Formulation

Our aim is to predict whether a patient will develop sepsis within the next $T$ hours based on their current and previously observed patient features. We make a prediction at each time along a patient’s trajectory, we call this the *real-time sepsis prediction* task.

We consider each of our three definitions **H1**, **H2** and **H3** with SOFA windows of (*x*, *y*) as one of $\{\left( 6,3 \right),\left( 12,6 \right),\left( 24,12 \right),\left( 48,24 \right)\}$. Where $x$ is the number of hours before t_suspicion_ in hours, and $y$ is the number of hours after $t$ _suspicion_ in hours, where we look for an increase in SOFA score of 2 or more points. For each choice of onset definition and SOFA window, we define the first sepsis onset time of the $i^{th}$ patient, denoted $t_{\mathrm{sepsis}}^{i}$. By convention, if the $i^{th}$ patient does not develop sepsis, $t_{\mathrm{sepsis}}^{i}=\infty$.

To be more specific, we let $x^{i}$ denote the static raw features of the $i^{th}$ patient, such as the demographic information. Let $x_{t}^{i}\in R^{d}$ denote the measurements taken of patient $i$ at time $t$. This includes all physiological and laboratory measured values (heart rate, blood pressure, glucose levels, and so on). The full list of the static features and the measurement variables (e.g., vital signs, laboratory results) used in our study is given in Table 6. The list of the derived features (e.g., the partial sum of SOFA) and the temporal features is provided in Table 7.

Table 6: Measurement variables used for extracting features.

| **Vital Signs** | **Laboratory Values** | **Demographics** | **Timestamps** |
| --- | --- | --- | --- |
| Heart rate | Base excess | Age | Admission time to hospital |
| Pulse oximetry (O2Sat) | Bicarbonate (${HCO}_{3}$) | Sex | Admission time to ICU |
| Temperature | Asparate Aminotransferase (AST) |  | Chart time |
| (Non-invasive) blood pressure systolic (SBP) | Fraction of inspired oxygen (${FiO}_{2}$) |  |  |
| Mean arterial pressure - estimated (MAP) | Arterial pH (pH) |  |  |
| (Non-invasive) blood pressure diastolic (DBP) | Arterial Partial pressure of ${CO}_{2}$ (${PaCO}_{2}$) |  |  |
| Respiratory rate | Oxygen saturation (${SaO}_{2}$) |  |  |
|  | Blood urea nitrogen (BUN) |  |  |
|  | Alkaline Phosphatase (ALP) |  |  |
|  | Calcium (${Ca}^{2+}$) |  |  |
|  | Chloride (${CI}^{-}$) |  |  |
|  | Creatinine |  |  |
|  | Bilirubin direct |  |  |
|  | Serum glucose |  |  |
|  | Lactic acid |  |  |
|  | Magnesium (${Mg}^{2+}$) |  |  |
|  | Phosphate (${Po}^{4+}$) |  |  |
|  | Potassium ($K^{+}$) |  |  |
|  | Total bilirubin |  |  |
|  | Troponin I |  |  |
|  | Hematocrit (Hct) |  |  |
|  | Hemoglobin (Hgb) |  |  |
|  | White blood cell count (WBC) |  |  |
|  | Partial thromboplastin time (PTT) |  |  |
|  | Fibrinogen |  |  |
|  | Platelets |  |  |

We define the training labels as

$$\begin{matrix} y_{t}^{i}=\left\{ \begin{matrix} 1 & \text{ if the patient develops sepsis within }T\text{ hours after time }t\text{,} \\ 0 & \text{ otherwise.} \end{matrix} \right. \end{matrix}$$

The goal is to train a model to predict the labels $y_{t}^{i}$ using the data $\left( x_{1}^{i},x_{2}^{i},...,x_{t}^{i} \right)$, that is, the data that has occurred at or before the time at which we wish to make a prediction. This is done for every time point and every patient.

We give an illustration of this in Figure 1. We wish to make a positive prediction at the time $t$ if the patient develops sepsis in the window $\left[ t,t+T \right]$ using data observed at or before time $t$.


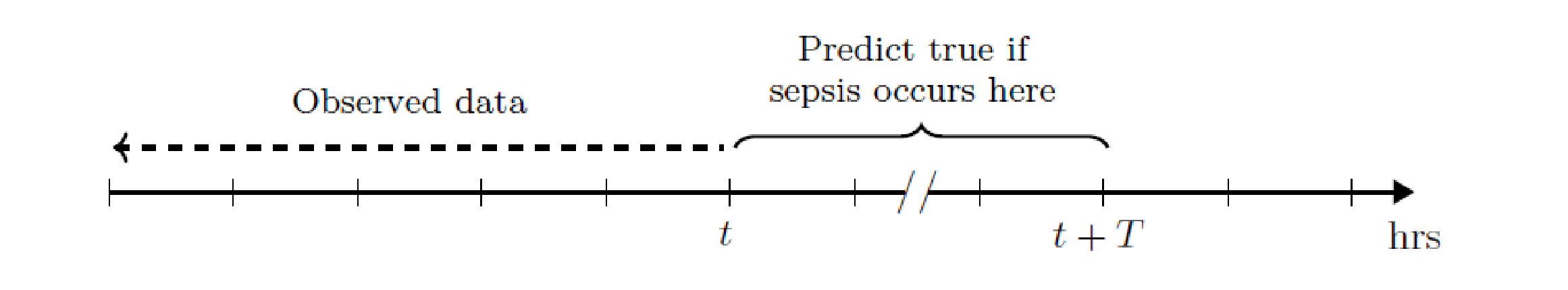


Figure 1: Illustration of real-time sepsis prediction task. For each time $t$ in a patient’s time series, the aim is to predict as true if the patient will develop sepsis in the next $T$ hours using the data up to and including the current time.

Table 7: Derived features from measurement variables.

| **Derived features** | **Derived from** |
| --- | --- |
| Partial SOFA | MAP, Bilirubin direct, Creatinine, Platelets |
| Shock index | Heart rate, SBP |
| BUN/Creatinine | BUN, Creatinine |
| Rolling counts | Laboratory Values, Temperature |
| Rolling maximums | Vital signs |
| Rolling minimums | Vital signs |
| Rolling 2nd-order moments | Vital signs |
| Signatures | Heart rate, SBP, MAP, Partial SOFA, BUN/Creatinine |

In Table 8 we give the expanded list of cultures we use to define a suspicion of infection in our sensitivity test. This is motivated by the fact that Singer et al. (5) and Seymour et al. (4) use bodily fluid cultures rather than just blood cultures.

Table 8: List of additional cultures added as part of our study for sensitivity analysis (with capitalization conventions based on the data in MIMIC-III).

| **Indicator of suspicion** | **Not indicator of suspicion** |
| --- | --- |
| ABSCESS | ARTHROPOD |
| ANORECTAL/VAGINAL CULTURE | Biopsy |
| ASPIRATE | Blood |
| BILE | Blood bag fluid |
| Blood (CMV AB) | BLOOD CULTURE - NEONATE |
| BLOOD CULTURE | BLOOD CULTURE (POST-MORTEM) |
| BLOOD CULTURE ( MYCO/F LYTIC BOTTLE) | BONE MARROW |
| Blood (EBV) | BONE MARROW - CYTOGENETICS |
| Blood (Malaria) | BRONCHIAL BRUSH |
| Blood (Toxo) | BRONCHIAL BRUSH - PROTECTED |
| BRONCHIAL WASHINGS | C, E, & A Screening |
| BRONCHOALVEOLAR LAVAGE | CORNEAL EYE SCRAPINGS |
| CATHETER TIP-IV | CRE Screen |
| CSF;SPINAL FLUID | DIALYSIS FLUID |
| Direct Antigen Test for Herpes Simplex Virus Types 1 & 2 | EAR |
| DIRECT ANTIGEN TEST FOR VARICELLA-ZOSTER VIRUS | EYE |
| FLUID RECEIVED IN BLOOD CULTURE BOTTLES | FECAL SWAB |
| FLUID WOUND | FLUID,OTHER |
| FOOT CULTURE | FOREIGN BODY |
| Immunology (CMV) | GASTRIC ASPIRATE |
| Influenza A/B by DFA | IMMUNOLOGY |
| Influenza A/B by DFA - Bronch Lavage | Isolate |
| Influenza A/B by DFA - Bronch Wash | MRSA SCREEN |
| JOINT FLUID | NAIL SCRAPINGS |
| Mini-BAL | NEOPLASTIC BLOOD |
| PERITONEAL FLUID | NOSE |
| PLEURAL FLUID | PERIPHERAL BLOOD LYMPHOCYTES |
| RAPID RESPIRATORY VIRAL ANTIGEN TEST | POSTMORTEM CULTURE |
| Rapid Respiratory Viral Screen & Culture | POST-MORTEM VIRAL CULTURE |
| SPUTUM | RECTAL - R/O GC |
| THROAT CULTURE | SCOTCH TAPE PREP/PADDLE |
| THROAT FOR STREP | SEROLOGY/BLOOD |
| TRACHEAL ASPIRATE | SKIN SCRAPINGS |
| TRANSTRACHEAL ASPIRATE | Staph aureus Screen |
| URINE | Stem Cell - Blood Culture |
| URINE,KIDNEY | STERILITY CULTURE |
| URINE,SUPRAPUBIC ASPIRATE | STOOL |
| VARICELLA-ZOSTER CULTURE | STOOL (RECEIVED IN TRANSPORT SYSTEM) |
| VIRAL CULTURE: R/O CYTOMEGALOVIRUS | SWAB |
| VIRAL CULTURE:R/O HERPES SIMPLEX VIRUS | SWAB, R/O GC |
|  | SWAB - R/O YEAST |
|  | THROAT |
|  | TISSUE |
|  | Touch Prep/Sections |
|  | TRACHEAL ASPIRATE |
|  | URINE,PROSTATIC MASSAGE |
|  | WORM |
|  | XXX |

# Appendix C: Feature extraction

We use three models in our analysis, namely, LGBM, LSTM, and CoxPHM. For a fair comparison, for all the models we use the same features $f_{t}^{i}$ from the observed measurement up to time $t$:

$$f_{t}^{i}=F\left( x^{i},x_{0}^{i},...,x_{t}^{i} \right),$$

where the feature extraction function $F$ extracts a variety of statistics, ranging from the patient demographics, denoted by $x^{i}$, the number of times each laboratory value has been measured, to nonlinear interactions of the physiological signal variables as they change in time.

The decision regarding which features to supply is a challenging one and has been the subject of much research [13]. In particular, the LGBM and Cox models do not process data sequentially and so temporal information used must be supplied to the models as additional features at each prediction time $t$. For example, if it is important to know the change in heart rate over the last six hours, we would need to explicitly supply the models with the temporal features to summarize the heart rate changes over the interval.

We choose the features which were used in the model that won the 2019 PhysioNet challenge on prediction of sepsis [16] as these represent the current gold standard for this problem. The proposed features include the measurable variables in Table 6, the derived features and the temporal features in Table 7 to capture the evolution of the physiological signals. For example, the signature features can describe rapid changes of vital signs. We refer the reader to the original paper for the full details of this mathematical construction.

For the LGBM and CoxPHM, we seek to predict $y_{t}^{i}$ from $f_{t}^{i}$ at each time point. The LSTM model processes data sequentially, passing information about the previous time to the next in order to learn temporal relations. Therefore, we use the $p$-lagged value of $f_{t}^{i}$, i.e., $\left( f_{t-p+1}^{i},\cdots,f_{t-1}^{i},f_{t}^{i} \right)$ as input to the model and allow the network to predict the $y_{t}^{i}$ at each time step.

# Appendix D: Models

## LGBM

Following the work in [14], we chose LightGBM (LGBM) as our representative of tree-based models. LGBM is a highly optimized gradient boosting tree algorithm that has proved to be particularly effective in sepsis prediction tasks. A (simple) decision tree [26], which is the foundation of all tree-based models, is a virtual upside-down tree with a decision rule at the root, from which subsequent decision rules spread out below. Gradient boosting is an ensemble method to combine weak learners to improve the predictive performance by using the stagewise optimization and iterative functional gradient descent [27]. As one of the gradient boosting algorithms based on tree weak learners, LGBM has the advantage of fast training and low memory cost [28]. As a non-parametric tree-based model, LGBM can be applied both to static data and time series data. The features of LGBM defined in Appendix C include the signature feature descriptions, which effectively summarize the temporal dependency of vital signs. As the sepsis detection is a classification task, we adopted the cross entropy as the impurity measure used in the loss function of LGBM.

## LSTM

Long short-term memory (LSTM) is a type of Recurrent Neural Network, which uses a looped structure to remember information from the past. These structures are very popular in problems where we want to retain past information/context, for example, in machine translation or time series. The strength of LSTM, in particular, is in capturing long term dependencies, and as such, is one of the most popular deep learning models. To do this, LSTM has the ability to add or remove information to the cell state, carefully regulated by a gate mechanism. The bidirectional LSTM [29] is a subclass of LSTM, which can use the input sequence information in the positive and negative time direction. The bidirectional LSTM (BLSTM) has empirically been shown to increase performance over the undirectional LSTM in various applications [30].

LSTM naturally takes a time series $x_{1:p}=\left( x_{1},\cdots,x_{p} \right)$ as an input to learn the temporal features of the input automatically. In our case, we choose the neural network, which is a BLSTM module [29] followed by a single dense layer and softmax activation function, to approximate the probability of developing sepsis within the next $T$ hours conditional on the given input.

*Undirectional LSTM* is composed of the input layer, the hidden layer and the output layer. In the hidden layer at time step $t$, the memory cell $c_{t}$ works as the core part of LSTM. In the memory cell, there are three gates denoted as the input gate $i_{t}$, the output gate $o_{t}$ and the forget gate $f_{t}$. Let $x_{t}$ be the input vector, $h_{t}$ be the output of the hidden layer, then the computation in the memory cell is shown in the following equations:

$$\begin{matrix} f_{t} & = & \sigma_{g}\left( W_{f}x_{t}+U_{f}h_{t-1}+b_{f} \right), \\ i_{t} & = & \sigma_{g}\left( W_{i}x_{t}+U_{i}h_{t-1}+b_{i} \right), \\ o_{t} & = & \sigma_{g}\left( W_{o}x_{t}+U_{o}h_{t-1}+b_{o} \right), \\ c_{t} & = & \sigma_{h}\left( W_{c}x_{t}+U_{c}h_{t-1}+b_{c} \right), \\ \tilde{c}_{t} & = & f_{t}\odot c_{t-1}+i_{t}\odot c_{t}, \\ h_{t} & = & o_{t}\odot\sigma_{h}\left( \tilde{c}_{t} \right), \end{matrix}$$

where $\{W_{f},W_{i},W_{o},W_{c},U_{f},U_{i},U_{o},U_{f}\}$ is the set of weight matrices; $\{b_{f},b_{i},b_{o},b_{c}\}$ is the set of bias terms; $\odot$ is the elementwise muliplication of two vectors; $\sigma_{g}$ is the sigmoid function and $\sigma_{h}$ is the hyperbolic tangent function. We denote the undirectional LSTM model on the input data $x_{1:p}$ as $LSTM\left( x_{1:p} \right)$. Here $\Theta:=\{U,W,b\}$ is the parameter set.

*Bidirectional LSTM* defines a map from $R^{d\times p}$ to $R^{e\times p}$ similarly to that of the undirectional LSTM. However, the bidirectional LSTM duplicates the LSTM layer in the network so as to have two layers side-by-side, then provides the input sequence ${(x}_{1},\cdots,x_{p})$ and the reversed input sequence ($x_{p},\cdots,x_{1}$) as input to the first layer and second order respectively. In formula, the bidirectional LSTM transforms $x_{1:T}$ to $o_{1:T}$ such that

$$z_{1:p}=LSTM\left( x_{1:p} \right),\vec{z}_{1:p}=LSTM\left( x_{p:1} \right),o_{1:p}=\left( \left[ z_{t},\vec{z}_{t} \right] \right)_{t=1}^{p}.$$

We used cross-entropy as the loss function to train LSTM, i.e.,

$$L\left( \hat{y}_{t}^{\left( i \right)},y_{t}^{\left( i \right)} \right)=-\left( \hat{y}_{t}^{\left( i \right)}\cdot log\left( y_{t}^{\left( i \right)} \right)+\left( 1-\hat{y}_{t}^{\left( i \right)} \right)\cdot log\left( 1-y_{t}^{\left( i \right)} \right) \right),$$

where $\hat{y}_{t}^{\left( i \right)}$ is the estimated probability of $i^{th}$ patient to develop sepsis within the next $T$ hours at time $t$.

## CoxPHM

Cox proportional hazards model (CoxPHM) is a semi-parametric method for investigating the relationship between covariables and the time to an event. CoxPHM is one of most popular models used in survival analysis. We will give an intuitive definition of the CoxPHM in a sliding window manner. First, let us define a few terms:

1. Hazard function $h\left( \tau\right)$: The risk of a patient becoming septic at time $\tau$. It can be estimated by $h\left( \tau\right)=h_{0}\left( \tau\right)e^{\beta X}$, where $h_{0}$ is the baseline hazard rate with time dependency, and the second component is a non-time dependent function of patient’s features $X$.
2. Survival function $S\left( \tau\right)$: The probability of a patient not having sepsis up to time $\tau$. It can be estimated by the following:

$$S\left( \tau\right)=e^{-\int_{0}^{\tau} h\left( t \right)dt}$$

3. The probability of a patient having sepsis by time $\tau$ is given by $P\left( \tau\right)=1-S\left( \tau\right)$ with $P\left( 0 \right)=0$.

Similarly to (8), we reinterpret the sepsis prediction problem as a survival analysis model with a sliding window. We consider the fixed right censoring window $C_{t}^{\left( i \right)}\equiv T$ and denote the time to sepsis of the $i^{th}$ patient at time $t$ by $\tau_{t}^{\left( i \right)}$, i.e.

$$\begin{matrix} \tau_{t}^{\left( i \right)}=\left\{ \begin{matrix} t_{\text{sepsis}}^{\left( i \right)}-t, & \text{if }\text{the }i^{th}\text{ patient is septic and }t_{\text{sepsis}}^{\left( i \right)}\in\left[ t,t+T \right]; \\ T+1, & \text{otherwise.} \end{matrix} \right. \end{matrix}$$

We then have the triplet $\left( X_{t}^{\left( i \right)},Y_{t}^{\left( i \right)},\tau_{t}^{\left( i \right)} \right)$, where $X_{t}^{\left( i \right)}$ is the covariables and $Y_{t}^{\left( i \right)}$ is the indicator of whether sepsis onset occurs during the window $\left[ t,t+T \right]$. Note that our samples of the triplet $\left( X_{t}^{\left( i \right)},Y_{t}^{\left( i \right)},\tau_{t}^{\left( i \right)} \right)_{t,i}$ may not be independent.

We used the built-in function CoxPHFitter from Python package lifelines to estimate the survival function $S\left( u\vee X_{t}^{\left( i \right)} \right)$, which is the probability that the $i^{\mathrm{th}}$ patient has not developed sepsis by time $u+t$. The parameters of CoxPHM are learned through maximising the log-likelihood of the hazard function $h\left( \tau\right)$ iteratively, we also applied $L_{1}$ regularization to prevent overfitting of the model. The sepsis risk score is calculated as $P\left( \tau_{t}^{\left( i \right)}\leq T \right)=1-S\left( T\vee X_{t}^{\left( i \right)} \right).$

# Appendix E: Model training

## Hyperparameter tuning

For each choice of $x,y$ and $T$, we conduct hyperparameter tuning. Hyperparameters for each model are tuned using $5$-fold cross-validation on the training set (85% of the total data). Once the best hyperparamters are identified the model is retrained on the full set of training data and predictions are made on the test data.

#### LGBM

The LGBM model is implemented in the python package LightGBM [28]. The best hyperparameters of the LGBM model are obtained via random search with cross validation. We apply sampling without replacement for random search, which is implemented in the Scikit-learn package [31]. Many hyperparameters can have an impact on model performance, generalization, and overfitting. For instance, we control the complexity of the tree model by tuning the number of leaves and the depth of tree. To combat overfitting, regularization parameters for $L_{1}$ or $L_{2}$ regularization along with *min_gain_to_split* are also included. For computational feasibility, we searched 500 different random configuration from a set of specified grid values. The full list of the hyperparameters and the grid that we searched over is given below.

Table 9: The full list of hyperparameters and the grid choices of the LGBM models.

| **Hyperparameter name** | **Searched range (grid)** |
| --- | --- |
| n_estimators | [40, 70, 100, 200, 400, 500, 800] |
| learning_rate | [0.05, 0.08, 0.1, 0.12] |
| colsample_bytree | [0.5, 0.6, 0.7, 0.8] |
| max_depth | [4, 5, 6, 7, 8] |
| num_leaves | [5, 10, 16, 20, 25, 36, 49] |
| reg_alpha | [0.001, 0.01, 0.05, 0.1, 0.5, 1, 2, 5, 10, 20, 50, 100] |
| reg_lambda | [0.001, 0.01, 0.05, 0.1, 0.5, 1, 2, 5, 10, 20, 50, 100] |
| min_split_gain | [0.0, 0.1, 0.2, 0.3, 0.4] |
| subsample | [5/12, 6/12, 7/12, 8/12, 9/12] |
| subsample_freq | [10, 20] |
| max_bin | [100, 250, 500, 1000] |
| min_child_samples | [49, 99, 159, 199, 259, 299] |
| min_child_weight | [20, 21, 22, 23, 24, 25, 26, 27, 28, 29, 30, 31, 32, 33, 34, 35, 36, 37, 38, 39, 40, 41, 42, 43, 44, 45, 46, 47, 48, 49] |

#### LSTM

LSTM model is implemented in PyTorch [32] and we use the Adam [33] optimizer with back-propagation to train model parameters. The LSTM model consists of one layer of bidirectional LSTM units followed by a single dense layer. All other hyperparameters, including the number of neurons for LSTM and dense layer, number of epochs, learning rate, batch sizes are carefully tuned with cross validation through a random search using the python package Tune [34]. We sampled a total of 80 configurations from the hyperparameter search space below.

Table 10: The full list of hyperparameters and the grid choices of the LSTM models.

| **Hyperparameter name** | **Search space** |
| --- | --- |
| hidden_channels | [16, 32, 48, 64] |
| linear_channels | [16, 32, 48, 64] |
| epochs | Sampled from Uniform({10, 11, …, 28, 29}) |
| lr | Sampled from Uniform(1e-4, 8e-4) |

#### CoxPHM

We implement hyperparmeter tuning of CoxPHM in the python package Tune [34]. The $L_{1}$ regularization and iterative step size are carefully chosen through random search to ensure the convergence of the model and obtain the best trained model. We sampled a total of 100 configurations from the hyperparameter search space below.

Table 11: The full list of hyperparameters and the grid choices of the CoxPHM models.

| **Hyperparameter name** | **Search space** |
| --- | --- |
| regularize | Sampled from Uniform(1e-4, 1e-3) |
| step_size | Sampled from Uniform(0, 0.2) |

# Appendix F: Numerical Results

In this section, we provide supplementary numerical results.


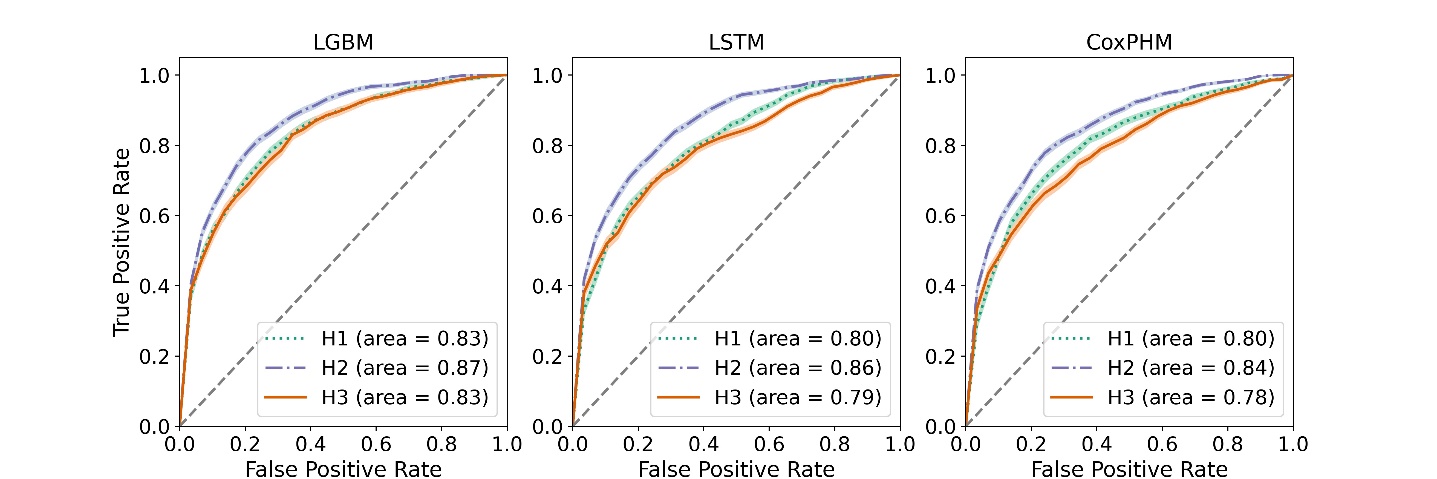


Figure 2: Receiver operating characteristic plots with 95% confidence interval of LGBM, LSTM and CoxPHM on the test set for the real-time sepsis prediction where $\left( x,y \right)=\left( 24,12 \right)$ and $T=6$.

*
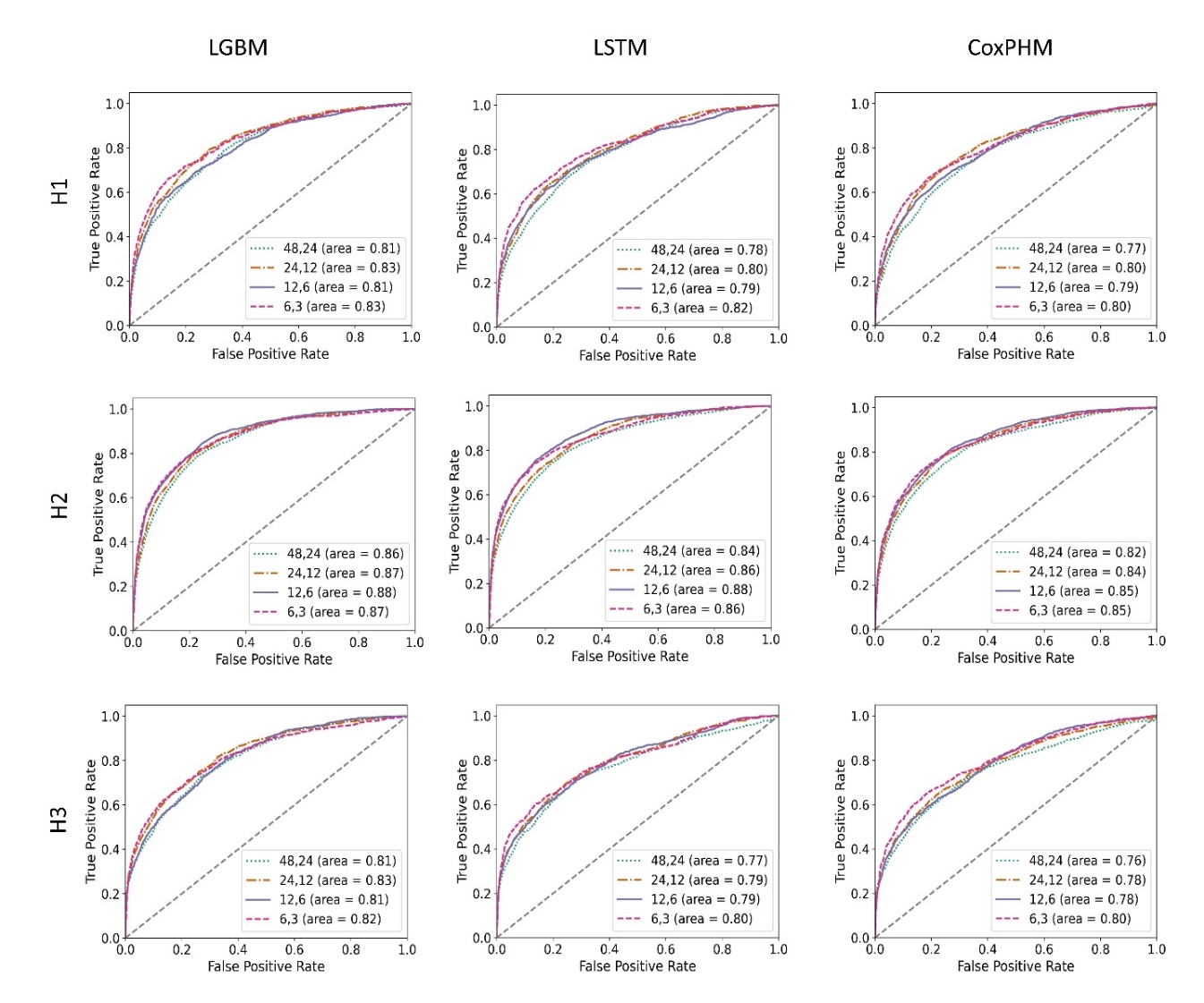
*

Figure 3: Receiver operating characteristic plots of all three models for the real-time prediction on the test data at different SOFA windows $\left( x,y \right)$ for prediction horizon $T=6$ and all three definitions.

Table 12: Summary of AUROC, specificity, accuracy (at fixed sensitivity level $85\%$ on the training set) of LGBM, LSTM and CoxPHM for the real-time prediction task for SOFA window $\left( x,y \right)=\left( 24,12 \right)$ and prediction horizon $T=6$. In the AUROC cell, the first number is the average AUROC, and the following interval is $95\%$ confidence interval of AUROC by bootstrapping.

|  | **AUROC(train)** | **Specificity** | **Accuracy** |
| --- | --- | --- | --- |
|  |
| **LGBM** | 0.883 [0.881,0.886] | 0.726 | 0.728 |
| **LSTM** | 0.833[0.829,0.837] | 0.606 | 0.611 |
| **CoxPHM** | 0.796 [0.791,0.801] | 0.536 | 0.543 |
|  |
| **LGBM** | 0.918 [0.916,0.920] | 0.818 | 0.819 |
| **LSTM** | 0.874 [0.871,0.876] | 0.721 | 0.725 |
| **CoxPHM** | 0.850 [0.847,0.853] | 0.667 | 0.673 |
|  |
| **LGBM** | 0.882 [0.879,0.886] | 0.718 | 0.720 |
| **LSTM** | 0.797 [0.793,0.802] | 0.509 | 0.514 |
| **CoxPHM** | 0.784 [0.779,0.790] | 0.483 | 0.488 |

Table 13: Summary of PPV and NPV (at fixed sensitivity level $85\%$ on the training set) of LGBM, LSTM, and CoxPHM for the real-time prediction task for SOFA window $\left( x,y \right)=\left( 24,12 \right)$ and prediction horizon $T=6$.

| **Model** | **PPV (test)** | **NPV (test)** | **PPV (train)** | **NPV (train)** |
| --- | --- | --- | --- | --- |
| **H1** | | | | |
| **LGBM** | 0.074 | 0.992 | 0.061 | 0.996 |
| **LSTM** | 0.046 | 0.992 | 0.043 | 0.995 |
| **CoxPHM** | 0.043 | 0.993 | 0.037 | 0.994 |
| **H2** | | | | |
| **LGBM** | 0.097 | 0.992 | 0.134 | 0.994 |
| **LSTM** | 0.084 | 0.992 | 0.091 | 0.993 |
| **CoxPHM** | 0.075 | 0.992 | 0.078 | 0.993 |
| **H3** | | | | |
| **LGBM** | 0.052 | 0.993 | 0.045 | 0.997 |
| **LSTM** | 0.030 | 0.994 | 0.026 | 0.995 |
| **CoxPHM** | 0.029 | 0.994 | 0.025 | 0.995 |

Table 14: AUROC/Specificity/Accuracy of different models for the real-time prediction on train set for different SOFA windows $\left( x,y \right)$ and prediction horizon $T$. The specificity and accuracy are calculated at 85% sensitivity level.

| $\mathbf{x,y}$ | $\mathbf{T}$ | **H1** | **H2** | **H3** |
| --- | --- | --- | --- | --- |
|  |  | LGBM | | |
| 48,24 | 12 | 0.869/0.721/0.726 | 0.900/0.768/0.772 | 0.877/0.697/0.701 |
|  | 8 | 0.867/0.713/0.717 | 0.905/0.781/0.784 | 0.878/0.711/0.713 |
|  | 6 | 0.870/0.720/0.723 | 0.904/0.785/0.787 | 0.883/0.728/0.730 |
|  | 4 | 00.872/0.722/0.724 | 0.903/0.781/0.783 | 0.886/0.734/0.735 |
| 24,12 | 12 | 0.877/0.730/0.734 | 0.916/0.809/0.811 | 0.874/0.705/0.708 |
|  | 8 | 0.880/0.727/0.730 | 0.920/0.820/0.821 | 0.878/0.703/0.706 |
|  | 6 | 0.883/0.726/0.728 | 0.918/0.818/0.819 | 0.882/0.718/0.720 |
|  | 4 | 0.887/0.740/0.742 | 0.918/0.819/0.820 | 0.887/0.733/0.734 |
| 12,6 | 12 | 0.885/0.744/0.747 | 0.931/0.850/0.850 | 0.882/0.734/0.736 |
|  | 8 | 0.892/0.758/0.760 | 0.937/0.863/0.863 | 0.882/0.717/0.719 |
|  | 6 | 0.896/0.762/0.764 | 0.936/0.862/0.862 | 0.891/0.733/0.735 |
|  | 4 | 0.901/0.772/0.773 | 0.936/0.860/0.860 | 0.895/0.739/0.740 |
| 6,3 | 12 | 0.903/0.798/0.799 | 0.933/0.864/0.864 | 0.888/0.751/0.753 |
|  | 8 | 0.911/0.799/0.799 | 0.939/0.876/0.876 | 0.900/0.768/0.769 |
|  | 6 | 0.915/0.813/0.813 | 0.940/0.877/0.876 | 0.906/0.788/0.789 |
|  | 4 | 0.918/0.815/0.815 | 0.943/0.882/0.882 | 0.910/0.795/0.796 |
|  |  | LSTM | | |
| 48,24 | 12 | 0.800/0.556/0.567 | 0.836/0.616/0.629 | 0.756/0.442/0.452 |
|  | 8 | 0.812/0.577/0.585 | 0.849/0.646/0.655 | 0.784/0.487/0.494 |
|  | 6 | 0.822/0.610/0.616 | 0.850/0.658/0.665 | 0.803/0.524/0.529 |
|  | 4 | 0.832/0.630/0.634 | 0.851/0.659/0.664 | 0.811/0.540/0.543 |
| 24,12 | 12 | 0.806/0.549/0.558 | 0.860/0.690/0.697 | 0.758/0.441/0.451 |
|  | 8 | 0.824/0.591/0.597 | 0.873/0.717/0.722 | 0.778/0.458/0.465 |
|  | 6 | 0.833/0.606/0.611 | 0.874/0.721/0.725 | 0.797/0.509/0.514 |
|  | 4 | 0.846/0.638/0.641 | 0.877/0.719/0.722 | 0.812/0.541/0.545 |
| 12,6 | 12 | 0.805/0.551/0.558 | 0.878/0.735/0.739 | 0.776/0.473/0.480 |
|  | 8 | 0.826/0.592/0.597 | 0.893/0.778/0.780 | 0.798/0.502/0.508 |
|  | 6 | 0.845/0.639/0.642 | 0.898/0.786/0.787 | 0.817/0.544/0.548 |
|  | 4 | 0.851/0.656/0.658 | 0.901/0.791/0.792 | 0.829/0.571/0.574 |
| 6,3 | 12 | 0.824/0.582/0.586 | 0.857/0.675/0.678 | 0.794/0.509/0.514 |
|  | 8 | 0.847/0.627/0.630 | 0.879/0.743/0.745 | 0.822/0.571/0.574 |
|  | 6 | 0.859/0.668/0.670 | 0.889/0.773/0.774 | 0.835/0.604/0.607 |
|  | 4 | 0.872/0.701/0.702 | 0.897/0.796/0.796 | 0.844/0.632/0.634 |
|  |  | CoxPHM | | |
| 48,24 | 12 | 0.760/0.476/0.490 | 0.821/0.582/0.597 | 0.755/0.441/0.451 |
|  | 8 | 0.774/0.503/0.513 | 0.833/0.613/0.624 | 0.774/0.468/0.475 |
|  | 6 | 0.782/0.522/0.530 | 0.835/0.623/0.631 | 0.785/0.482/0.488 |
|  | 4 | 0.787/0.535/0.541 | 0.834/0.613/0.620 | 0.794/0.506/0.510 |
| 24,12 | 12 | 0.772/0.481/0.492 | 0.836/0.623/0.634 | 0.754/0.432/0.442 |
|  | 8 | 0.788/0.516/0.524 | 0.848/0.656/0.664 | 0.773/0.467/0.474 |
|  | 6 | 0.796/0.536/0.543 | 0.850/0.667/0.673 | 0.785/0.483/0.488 |
|  | 4 | 0.801/0.547/0.552 | 0.850/0.659/0.664 | 0.793/0.496/0.500 |
| 12,6 | 12 | 0.774/0.486/0.494 | 0.857/0.670/0.676 | 0.767/0.466/0.473 |
|  | 8 | 0.793/0.518/0.524 | 0.872/0.728/0.732 | 0.786/0.497/0.503 |
|  | 6 | 0.804/0.546/0.551 | 0.876/0.743/0.746 | 0.799/0.518/0.522 |
|  | 4 | 0.813/0.572/0.575 | 0.876/0.733/0.735 | 0.809/0.552/0.555 |
| 6,3 | 12 | 0.797/0.529/0.534 | 0.844/0.636/0.640 | 0.794/0.518/0.523 |
|  | 8 | 0.819/0.576/0.580 | 0.865/0.701/0.704 | 0.817/0.572/0.575 |
|  | 6 | 0.830/0.606/0.608 | 0.872/0.734/0.736 | 0.828/0.600/0.603 |
|  | 4 | 0.838/0.621/0.623 | 0.879/0.739/0.740 | 0.836/0.609/0.611 |

Table 15: AUROC/Specificity/ Sensitivity Accuracy of different models for the real-time prediction on test set. for different SOFA windows $\left( x,y \right)$ and prediction horizon $T$. The specificity, accuracy and sensitivity are calculated using the threshold, which was fixed at 85% sensitivity level on the training set.

| $\mathbf{x,y}$ | $\mathbf{T}$ | **H1** | **H2** | **H3** |
| --- | --- | --- | --- | --- |
|  |  | **LGBM** | | |
| **48,24** | **12** | 0.787/0.719/0.707/0.708 | 0.840/0.778/0.751/0.752 | 0.759/0.696/0.690/0.690 |
|  | **8** | 0.796/0.722/0.703/0.704 | 0.853/0.786/0.764/0.765 | 0.794/0.733/0.705/0.705 |
|  | **6** | 0.807/0.733/0.710/0.711 | 0.857/0.788/0.771/0.772 | 0.806/0.736/0.719/0.720 |
|  | **4** | 0.812/0.741/0.715/0.716 | 0.860/0.792/0.769/0.769 | 0.817/0.735/0.730/0.730 |
| **24,12** | **12** | 0.806/0.745/0.719/0.720 | 0.857/0.748/0.798/0.795 | 0.785/0.738/0.693/0.694 |
|  | **8** | 0.823/0.767/0.722/0.723 | 0.866/0.757/0.808/0.806 | 0.815/0.777/0.694/0.696 |
|  | **6** | 0.832/0.784/0.723/0.725 | 0.869/0.761/0.808/0.806 | 0.829/0.768/0.713/0.714 |
|  | **4** | 0.842/0.779/0.736/0.737 | 0.875/0.771/0.809/0.808 | 0.840/0.773/0.728/0.729 |
| **12,6** | **12** | 0.765/0.653/0.733/0.731 | 0.858/0.700/0.839/0.834 | 0.757/0.637/0.719/0.717 |
|  | **8** | 0.787/0.664/0.751/0.749 | 0.879/0.727/0.853/0.849 | 0.792/0.714/0.710/0.710 |
|  | **6** | 0.807/0.696/0.755/0.754 | 0.882/0.725/0.855/0.851 | 0.813/0.720/0.726/0.726 |
|  | **4** | 0.818/0.713/0.763/0.763 | 0.886/0.741/0.853/0.851 | 0.820/0.712/0.732/0.731 |
| **6,3** | **12** | 0.818/0.713/0.763/0.763 | 0.886/0.741/0.853/0.851 | 0.820/0.712/0.732/0.731 |
|  | **8** | 0.818/0.682/0.793/0.792 | 0.860/0.689/0.869/0.866 | 0.808/0.698/0.761/0.761 |
|  | **6** | 0.835/0.708/0.810/0.809 | 0.874/0.708/0.870/0.868 | 0.821/0.703/0.783/0.782 |
|  | **4** | 0.841/0.721/0.810/0.810 | 0.889/0.718/0.878/0.877 | 0.838/0.732/0.788/0.788 |
|  |  | **LSTM** | | |
| **48,24** | **12** | 0.755/0.802/0.546/0.557 | 0.824/0.849/0.625/0.638 | 0.727/0.819/0.421/0.432 |
|  | **8** | 0.766/0.803/0.552/0.560 | 0.837/0.834/0.665/0.672 | 0.746/0.816/0.466/0.474 |
|  | **6** | 0.784/0.795/0.592/0.598 | 0.837/0.839/0.649/0.656 | 0.767/0.815/0.503/0.509 |
|  | **4** | 0.786/0.796/0.606/0.610 | 0.842/0.833/0.674/0.678 | 0.784/0.816/0.507/0.511 |
| **24,12** | **12** | 0.770/0.811/0.551/0.560 | 0.842/0.816/0.694/0.700 | 0.740/0.801/0.470/0.479 |
|  | **8** | 0.788/0.805/0.580/0.586 | 0.852/0.810/0.714/0.718 | 0.775/0.862/0.442/0.450 |
|  | **6** | 0.805/0.811/0.596/0.601 | 0.856/0.813/0.718/0.721 | 0.793/0.831/0.524/0.529 |
|  | **4** | 0.832/0.845/0.632/0.636 | 0.856/0.819/0.719/0.721 | 0.816/0.843/0.557/0.561 |
| **12,6** | **12** | 0.755/0.789/0.548/0.554 | 0.851/0.802/0.736/0.738 | 0.734/0.793/0.488/0.495 |
|  | **8** | 0.774/0.775/0.594/0.598 | 0.876/0.814/0.778/0.779 | 0.767/0.826/0.513/0.518 |
|  | **6** | 0.792/0.773/0.638/0.640 | 0.877/0.800/0.784/0.785 | 0.790/0.828/0.563/0.567 |
|  | **4** | 0.816/0.795/0.652/0.653 | 0.876/0.791/0.791/0.791 | 0.804/0.824/0.593/0.595 |
| **6,3** | **12** | 0.772/0.777/0.591/0.594 | 0.818/0.769/0.673/0.675 | 0.745/0.767/0.512/0.516 |
|  | **8** | 0.794/0.784/0.631/0.633 | 0.850/0.793/0.743/0.743 | 0.777/0.779/0.577/0.580 |
|  | **6** | 0.818/0.782/0.678/0.679 | 0.864/0.795/0.772/0.773 | 0.796/0.793/0.609/0.611 |
|  | **4** | 0.831/0.787/0.706/0.707 | 0.875/0.801/0.797/0.797 | 0.815/0.806/0.635/0.636 |
|  |  | **CoxPHM** | | |
| **48,24** | **12** | 0.738/0.825/0.481/0.495 | 0.814/0.842/0.590/0.604 | 0.718/0.799/0.445/0.455 |
|  | **8** | 0.755/0.824/0.508/0.519 | 0.825/0.845/0.619/0.629 | 0.741/0.809/0.472/0.480 |
|  | **6** | 0.768/0.832/0.526/0.534 | 0.825/0.845/0.626/0.633 | 0.758/0.818/0.491/0.497 |
|  | **4** | 0.775/0.840/0.538/0.544 | 0.823/0.845/0.615/0.621 | 0.770/0.822/0.516/0.520 |
| **24,12** | **12** | 0.766/0.839/0.496/0.508 | 0.830/0.829/0.631/0.641 | 0.736/0.819/0.438/0.448 |
|  | **8** | 0.786/0.845/0.528/0.537 | 0.843/0.831/0.663/0.669 | 0.764/0.829/0.470/0.477 |
|  | **6** | 0.799/0.850/0.546/0.553 | 0.844/0.830/0.672/0.677 | 0.780/0.840/0.490/0.496 |
|  | **4** | 0.807/0.857/0.557/0.562 | 0.843/0.827/0.661/0.665 | 0.796/0.870/0.499/0.504 |
| **12,6** | **12** | 0.749/0.801/0.503/0.511 | 0.836/0.808/0.675/0.680 | 0.734/0.796/0.475/0.482 |
|  | **8** | 0.772/0.811/0.534/0.539 | 0.854/0.805/0.729/0.731 | 0.763/0.816/0.509/0.515 |
|  | **6** | 0.789/0.819/0.557/0.561 | 0.855/0.799/0.743/0.745 | 0.783/0.828/0.541/0.545 |
|  | **4** | 0.801/0.829/0.578/0.581 | 0.852/0.800/0.733/0.734 | 0.795/0.823/0.565/0.568 |
| **6,3** | **12** | 0.753/0.777/0.533/0.537 | 0.806/0.769/0.642/0.644 | 0.747/0.773/0.524/0.528 |
|  | **8** | 0.786/0.787/0.582/0.584 | 0.836/0.791/0.705/0.706 | 0.783/0.784/0.577/0.579 |
|  | **6** | 0.803/0.793/0.611/0.613 | 0.848/0.796/0.739/0.739 | 0.801/0.795/0.599/0.601 |
|  | **4** | 0.817/0.809/0.626/0.627 | 0.858/0.801/0.744/0.745 | 0.816/0.802/0.623/0.624 |

Table 15: Summary of AUROC, specificity, accuracy (at fixed sensitivity level $85\%$ on the training set) of LGBM, LSTM and CoxPHM for the patient classification for SOFA window $\left( x,y \right)=\left( 24,12 \right)$ and prediction horizon $T=6$. In the AUROC (train/test) cell, the first number is the average AUROC, and the following interval is $95\%$ confidence interval of AUROC by bootstrapping. In the cells of specificity and accuracy, the first number and the second number (in the bracket) represent on the metrics on the test set and training set resp.

|  | **AUROC (train)** | **AUROC (test)** | **Specificity** | **Sen(test)** | **Accuracy** |
| --- | --- | --- | --- | --- | --- |
|  | **H1** | | | | |
| **LGBM** | 0.778 [0.764,0.791] | 0.742 [0.711,0.771] | 0.431(0.447) | 0.846 | 0.500(0.506) |
| **LSTM** | 0.744 [0.729,0.759] | 0.724 [0.690,0.753] | 0.356(0.396) | 0.864 | 0.439(0.461) |
| **CoxPHM** | 0.689 [0.674,0.705] | 0.675 [0.638,0.710] | 0.304(0.317) | 0.838 | 0.392(0.394) |
|  | **H2** | | | | |
| **LGBM** | 0.854 [0.845,0.864] | 0.814 [0.786,0.841] | 0.647(0.659) | 0.828 | 0.684(0.699) |
| **LSTM** | 0.807 [0.796,0.818] | 0.797 [0.772,0.821] | 0.536(0.548) | 0.855 | 0.601(0.611) |
| **CoxPHM** | 0.792 [0.782,0.803] | 0.783 [0.759,0.807] | 0.527(0.515) | 0.848 | 0.593(0.585) |
|  | **H3** | | | | |
| **LGBM** | 0.803 [0.787,0.819] | 0.765 [0.732,0.796] | 0.470(0.506) | 0.810 | 0.513(0.544) |
| **LSTM** | 0.758 [0.740,0.776] | 0.760 [0.720,0.793] | 0.399(0.403) | 0.875 | 0.459(0.452) |
| **CoxPHM** | 0.721 [0.702,0.741] | 0.711 [0.666,0.748] | 0.319(0.348) | 0.875 | 0.390(0.404) |

# Appendix G: Predicted Sepsis Onset Time

## One can use the real-time prediction model to infer other clinical outcomes on the patient level. Here we considered a *patient-level classification task*, that is, whether our method successfully flagged each patient at some time prior to the sepsis onset time $\boldsymbol{t}_{\boldsymbol{sepsis}}$. We classified a patient as “sepsis advance notified” if they were flagged as septic by the real-time prediction method at least once before the censored time (when sepsis-III criteria are met, or the patient is no longer in ICU). We computed the corresponding AUROC as a test metric to quantify the performance of sepsis classification at the patient-level. For “sepsis advance notified” patients we investigated the difference between the predicted and actual sepsis onset time.

We fixed a new threshold to ensure 85% sensitivity for the patient-level classification problem on the training set. For each sepsis onset definition, LGBM and LSTM models outperformed the CoxPHM on both training and test sets. The AUROC of LGBM achieves the highest of the three models for all definitions. The corresponding results on the test set can be found in Figure 4.


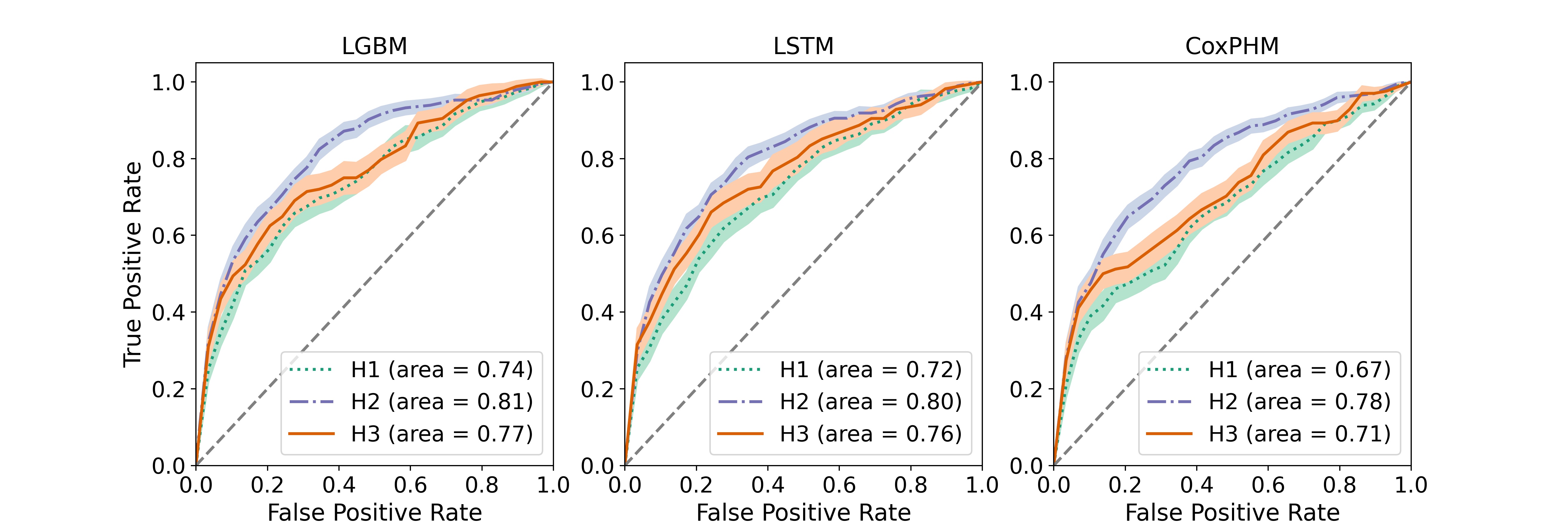


Figure 4: Receiver operator characteristic plots with bootstrapped 95% confidence interval of LGBM, LSTM and CoxPHM for the patient-level sepsis classification on the test set with SOFA window $\left( 24,12 \right)$ and prediction horizon $T=6$.

We see that all performance metrics are highest when we train the models under definition **H2** and lowest under **H1** with Table 12 showing the difference between $7-11$% for a fixed model.


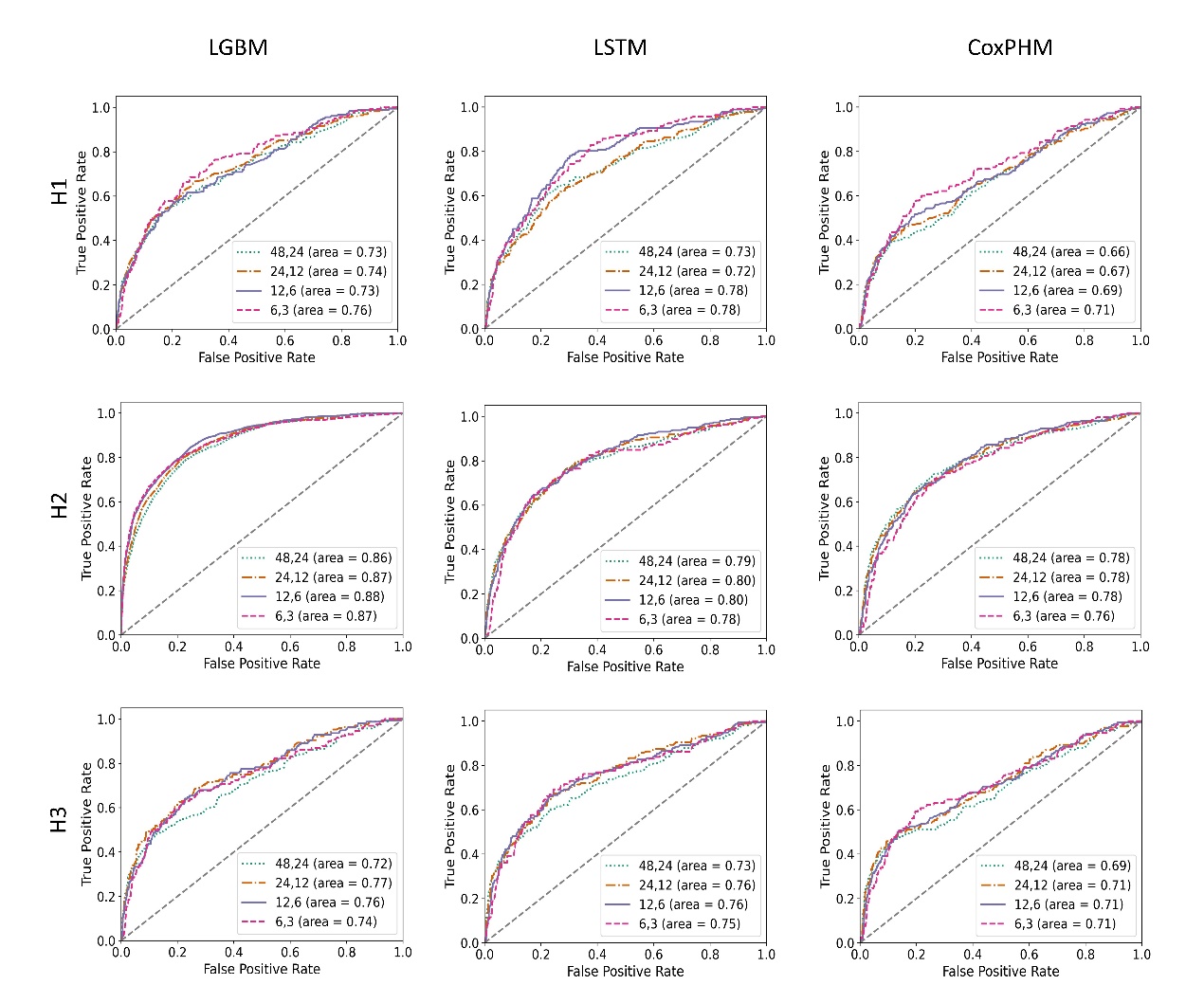


Figure 5: Receiver operator characteristic plots of LGBM, LSTM and CoxPHM for the patient-level sepsis classification on the test set with varying SOFA window and prediction horizon $T=6$.

There was variability between sepsis onset definitions and how far in advance the models were able to detect impending sepsis. Figure 6 and Figure 7 show clear variability in the predicted sepsis onset time with respect to the definitions.


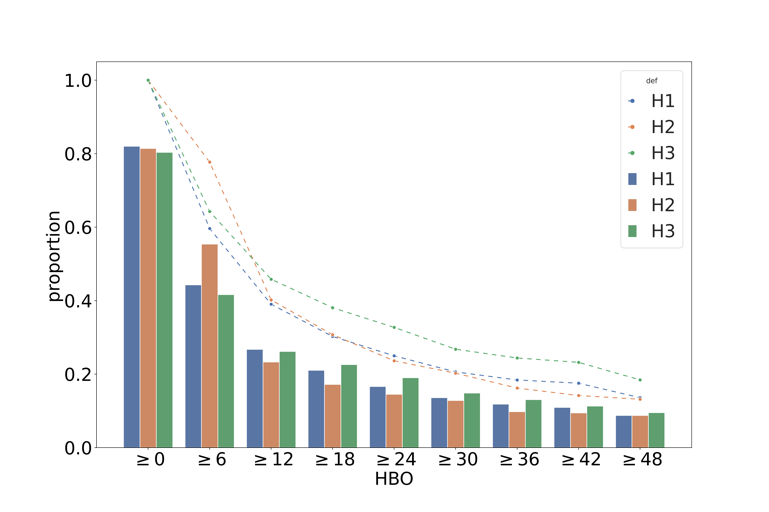


Figure 6: Bars: Of those patients who ultimately develop sepsis (under each definition), the proportion who have been given a warning flag, in terms of the hours before the onset of sepsis (HBO). Lines: the proportion of these patients who have been admitted to ICU, in terms of the hours before onset.

In Figure 6, we consider how many hours before the onset of sepsis a patient is flagged, given that they will develop sepsis. We would hope that our model flags patients six hours before the onset of sepsis, and does not flag patients more than six hours before onset. We see that a significant number of patients are flagged much earlier than the desired $T=6$ hours before onset. This is particularly the case when we take into account that many patients are not in ICU more than six hours before developing sepsis (as indicated by the dashed lines, which indicate the proportion of ultimately-septic patients who were in ICU at a certain time before the onset of sepsis).


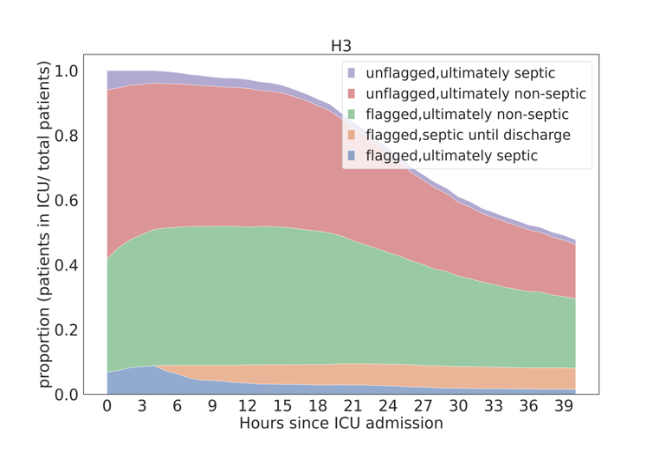

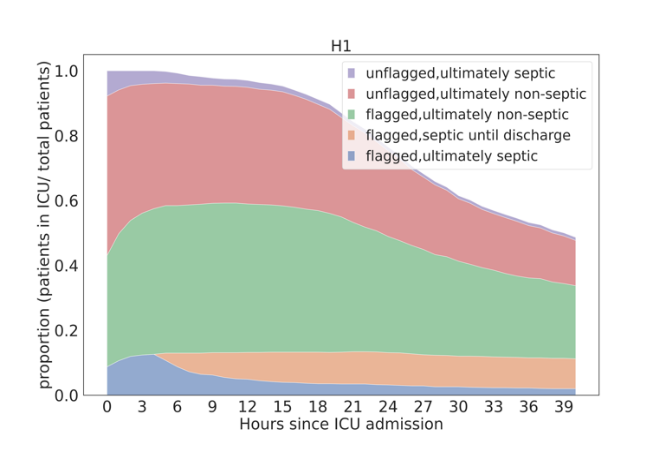

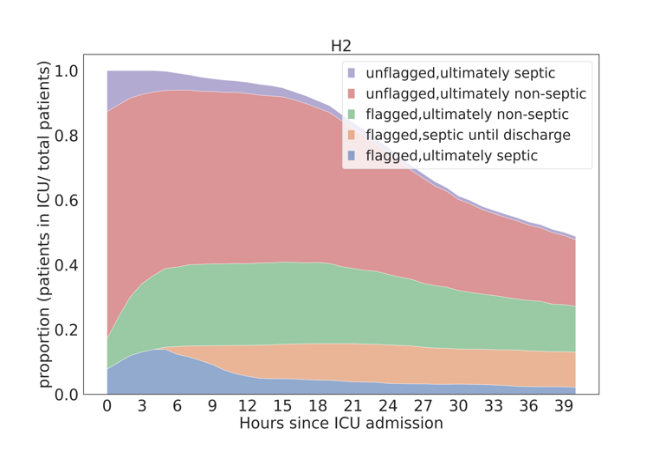


Figure 7: The proportions of patients as classified by whether they ultimately develop sepsis, have already developed sepsis, and have been flagged, in terms of the time since admission to ICU, for each definition via LGBM. The results shown are for the test data, using the LGBM model with SOFA window (24, 12) and prediction horizon T = 6. The probability threshold to determine septic label is chosen to fix the proportion of patients flagged before deterioration (in the training set) at 85%.

In Figure 7, we consider a different view, considering the status of patients after their initial ICU admission, using different definitions and the LGBM model for sepsis prediction. We see that a large number of patients who do not ultimately develop sepsis are unflagged (red), but then become flagged over the first five hours (green). Very few ultimately-septic patients are unflagged six hours after admission (purple). Of those patients who develop sepsis, most do between 6 and 9 hours after admission (yellow), with very few new sepsis cases observed more than 24 hours after admission. We also see that the number of patients who remain in ICU decreases rapidly after 15 hours, particularly among those patients who were flagged as septic but did not develop sepsis. After 36 hours in ICU, approximately 70% of remaining patients are flagged as septic, while approximately 25% have developed sepsis (most of whom developed it in the first 15 hours).

We can also see that the different definitions result in substantially different cohorts. The most restrictive exclusion criteria (under definition **H3**) result in a significant reduction in the number of sepsis cases. Under definition **H2**, we see a significant reduction in the proportion of patients initially flagged (blue + green areas), but a higher proportion of patients who develop sepsis without being flagged (purple area). As we saw in Table 2 of the main paper, this improved performance under definition **H2** largely disappears when considering the more restrictive exclusion criteria under **H3**.
